# The role of digitalization in business and management: a systematic literature review

**DOI:** 10.1007/s11846-023-00647-8

**Published:** 2023-03-28

**Authors:** Esther Calderon-Monge, Domingo Ribeiro-Soriano

**Affiliations:** 1 Department of Economics and Business Administration, Faculty of Economy and Business Studies, University of Burgos, Burgos, Spain; 2 IUDESCOOP-Universitat de València, València, Spain

**Keywords:** Accounting, Digitalization, Management, Marketing, Finance, Systematic literature review, M1, M3, M4

## Abstract

Digitalization is a powerful engine for economic growth in the world. In 2018, digitally transformed firms represented 13.5 billion US dollars of global GDP and, towards the end of 2023, they are expected to represent 53.3 billion US dollars, over half of the general nominal GDP (Statista, Nominal GDP driven by digitally transformed and other enterprises worldwide 2018–2023. https://www.statista.com/statistics/1134766/nominal-gdp-driven-by-digitally-transformed-enterprises/, 2022). The main objective of this study is to provide information (highlighting principal research topics and research agendas) from the literature on state-of-the-art digitalization within firms through a Systematic Literature Review (SLR). In all, 119 review articles on the most mature functional areas of the firm are analyzed: management, marketing, and finance and accounting, published in the WOS over the period 2018-April 2022. In this study, key relevant tendencies are identified in the most mature areas of the firm, which are the impact of digital technologies on the analysis of consumer behavior; digitalization and green innovation within organizations; and blockchain technology applied to financial services. The main contributions of this work are as follows: (1) to provide the most complete and up-to-date review of digitalization from a global perspective, summarizing the current state of knowledge within an integrated framework; (2) to reduce the complexity of digitalization by offering structure and clarity; and (3) to offer links between digitalization and established points of view in the literature on management, marketing, finance, and accounting. The novelty of this paper is centered on a joint analysis of digitalization, digital transformation, and digital technologies, taking into account the most mature functional areas of the firm.

## Introduction

Digitalization and the innovation that it drives are changing organizations, institutions, and society in general (Kraus et al. 2021). Centering attention on organizations, digitalization is provoking disruptive changes within firms and their immediate business environment, accelerating the obsolescence of the current business model (Wirtz et al. 2022). Digital technologies play a central role in the creation and the reinforcement of disruptions that take place in society and the levels of industry (Aström et al. 2022). Faced with these disruptions, organizations design strategic responses and they use digital technologies to alter pathways to value creation in which they had previously trusted for continued competitiveness. To do so, they should implement structural changes and overcome the obstacles to Digital Transformation (DT) that hinder their efforts (Hess et al. 2016). These disruptions trigger strategic responses among the organizations that occupy a central place in the literature on DT.

Digital technologies also alter consumer expectations and behavior in disruptive ways (Vial 2019). When employing these technologies, the consumer assumes the role of an active participant in the dialogue between an organization and its stakeholders (Yeow et al. 2017). Consequently, the clients no longer see themselves as so externally dependent on a firm with which they can negotiate (Sia et al. 2016) and their expectations multiply with regard to the services that the firm could offer to them. The implementation of Information Technology (IT) and organizational transformation (Gray et al. 2013) shifts the attention of the firm and its supply chain and directs it towards customers with digital connections.

Digital technologies can also be disruptive when altering the competitive landscape. The use of platforms (*i*.*e*., P2P in finance) have redefined existing markets giving rise to the sharing economy (Richter et al. 2017) and facilitating the exchange of digital goods and services. Competition is no longer only physical and is converted into a virtual domain where information flows are quicker and less restrictive than in the physical world and the former barriers to entry become less meaningful. This behavior can be seen in the music industry where physical goods have been replaced by subscription services for music offered by firms that had never before formed part of the industry. Companies can therefore use digital technologies both to launch new processes and to improve internal processes with their supply chains and their environment, as much as to develop new business models (Bouncken et al. 2021).

Given the growing importance of digitalization and its effects on firms, the main objective of this study is to improve our understanding of digitalization in the most mature areas of the firm, in order to identify gaps and future lines of investigation. To do so, we performed a Systematic Literature Review (SLR) for two reasons: (1) to use an adapted protocol to guide the data curation and analysis process, and to ensure objectivity (Kraus et al. 2022); and (2) a high-quality review paper has to follow an established methodology for the systematic selection and analysis of papers, and should periodically cover different fields to identify the latest developments (Snyder 2019). As with the recent SLR of Chaudhary et al. (2021), and others listed in the bibliography of this paper, this study encompasses three distinct stages: specifying the objective of the research; outlining the research protocol; and, finally, reporting the findings. Having specified the main objective of this study, the following Research Questions (RQ) are set out below:

- *RQ1*: How has the literature on digitalization developed up until 2022 in relation to different areas of the firm: management, marketing, and finance and accounting?
- *RQ2*: What are the principal research topics in relation to digitalization within different areas of the firm: management, marketing, and finance and accounting?
- *RQ3*: What is the research agenda in relation to digitalization within different areas of the firm: management, marketing, and finance and accounting?

These research questions are addressed through the SLR approach of Tranfield, Denver, and Smart (2003) to identify and to consolidate existing knowledge for its critical analysis and to generate ideas (Ribeiro-Navarrete et al. 2021). It is an established approach for the generation of robust, reliable, and replicable findings (Chaudhary et al. 2021). In addition, it is well suited to our objective, because its purpose is to consolidate the development of knowledge by identifying gaps for future research in mature areas (Vrontis et al. 2020; Chaudhary et al. 2021). We therefore chose management, marketing, and finance and accounting as mature areas of the firm where digitalization should be reviewed.

After applying the aforementioned research protocol that will be explained in Sub-Sect. 3.1, we will respond to the RQs. In response to RQ1, we generated descriptive statistics from peer-reviewed research articles selected by outlining the research context. We approached RQ2 by employing content analysis to outline key themes that emerged from the articles that were reviewed. Themes that researchers conducting SLR have recently often identified through the use of content analysis (Skare et al. 2022), to understand the intellectual structure of the field (Chaudhary et al. 2021). Finally, we responded to RQ3 by discovering gaps and avenues for future research.

The contribution of this paper to the literature is through a systematic review of the findings of 119 review articles published in academic journals on digitalization within each of the aforementioned areas. In particular, the theoretical contributions of this work are as follows: (1) to provide the most complete and updated review of digitalization from a global perspective, summarizing the current state of knowledge within an integrated framework; (2) to reduce the complexity of digitalization by offering structure and clarity; and (3) to offer links between digitalization and established points of view in the literature on management, marketing, finance and accounting. In addition, this literature review will help scholars to develop novel empirical studies of interest in subsequent investigations (Post et al. 2020), and to propose new routes and opportunities within this field.

## Theoretical framework

Digital tools such as Social, Mobile, Analytics, and Cloud (SMAC) technologies are driving digitalization (Teubner and Stockhinger 2020), and offering opportunities to change the way in which firms work (Aström et al. 2022). In accordance with Chan et al. (2022), social networks give market visibility to a firm and establish links with their actors; mobile networks also connect different actors within the business eco-system, and offer learning and continuous access to information at any time and place. The cloud brings accessibility, storage, and relevant information exchange, work-flow monitoring, and remote collaboration. Finally, analytics facilitates understanding of business and client needs together with the identification of opportunities and market trends, and the recommendation and provision of services and personalized communications. Digital technologies are therefore easily available to the firm and can improve its effectiveness in profitable ways (Chan et al. 2022), provided their introduction is accompanied by innovative business models or transformations of the traditional model (Aström et al. 2021). Nevertheless, investigation must continue on the impact of new technologies for the decision-making process of the firm (Troise et al. 2022) and consumer privacy (Quach et al. 2022).

Although SMAC technologies drive digitalization, it is not merely a technical advance, but it is also an economic and a social one too (Legner et al. 2017). Digitalization refers to the interaction between digital technologies and both the social the institutional processes converting these technologies into infrastructural technologies and impacting on society and the economy (Teubner and Stockhinger 2020), thereby advancing communication, mobility, speed, virtualization, the disappearance of frontiers, interconnections, market transparency, and competition. However, the technical process of coding analogic information in a digital format, which means that the digitalized content is programmable, traceable, and communicable is known as digitization (Yoo et al. 2010a, b). It is a technical phenomenon and must not be confused with digitalization, because it includes fewer integral changes. Nevertheless, digitalization is found at some point between digitization and DT. DT implies immense organizational changes driven by digital technologies and, in consequence, profound alterations in business strategies and routines (Alzamora et al. 2021). However, digitalization is associated with important changes within sociotechnical structures (Yoo et al. 2010a, b), which are reconfigured through questioning the assumptions underlying the design and the use of digital technologies (Thorseng and Grisot 2017). Therefore, DT is naturally connected to the topic of organizational change, viewed as a ‘difference in form, quality, or state over time in an organizational entity’ (Van de Ven and Poole 1995, p. 512). For greater knowledge of DT in the field of management, see Hanelt et al. (2021).

When digital technologies are installed in an organization, they interact with organizational and managerial characteristics, specifically with the strategy and the legacy of an organization, as well as with the resources, the processes, the values, and the culture of an organization (Dewan et al. 2003), without forgetting positive attitudes towards change and technology (Dery et al. 2017). All these organizational antecedents are integrated and interact with environmental antecedents and the characteristics of the country, the industry, and the consumers. These characteristics include the legal conditions and the infrastructure of a country, as well as its regulatory frameworks and interventions (Cortet et al. 2016), and the dynamics of an industry driven by technology, which includes the changing technological panorama of a country (Alos-Simo et al. 2017). The above in no way ensures the success of many digital transformations within firms. In a recent study, Witschel et al. (2022) demonstrated that the innovation of the business model is an effective way of continuing to be competitive in the digital era. To do so, these authors defended the role that dynamic capabilities play in the innovation of the business model, as well as contextual factors, leadership and business mentalities. Moreover, Wen et al. (2022) affirmed that manufacturing firms with greater viability are more adaptable to DT and tend to implement differentiated competitive strategies, for which reason they concluded that the effect of incentivizing innovation is greater for firms of higher viability. The antecedents of DT (digital orientation, digital intensity, and digital maturity) were also analyzed to understand their influence on the financial success of firms (Nasisri et al. 2022). These authors maintained that digital orientation and digital intensity in themselves contributed nothing to the financial success of firms; and digital maturity acted as a mediator between digital orientation and the financial success of firms, and between digital intensity and the financial success of firms. DT, therefore, presented important opportunities for firms, but also for entrepreneurs. In the case of digital entrepreneurship, Chatterjee et al. (2022) provided evidence that perceived utility, perceived ease of use, and willingness to introduce strong and significant changes all affected digital entrepreneurship.

In addition, a DT is shaped by the characteristics of the consumer, in particular, the demand of the digital consumer. Consumers place increasing trust in digital technologies throughout their daily lives and personal interactions (Brynjolfsson et al. 2013) and expect ubiquitous access to virtual resources (Benlian et al. 2018). Both the appearance of digital technologies and their diffusion have led to greater data availability (on these aspects, see Vial 2019, p.123), which has in turn increased the importance of automated learning and the analysis of data for organizations (Weichert 2017). These data enable firms to offer services that better respond to the needs of their clients and to complete processes in more efficient ways for their own competitive advantage. Thus, some firms use social networks such as Twitter and Facebook to institute customer care operations and then use the data that are generated from these interactions to appeal to client sentiment in real time. Finally, the digital industrial transformation, known as the fourth industrial revolution or Industry 4.0 implies a change of paradigm from a hyperconnected and centralized manufacturing ecosystem to a decentralized one (Li et al. 2019). Under Industry 4.0, intelligent physical objects, decentralized subsystems, and even human components are perfectly integrated within an interoperable, hyperconnected, and decentralized production system (see Hodapp and Hanelt 2022) that is capable of adapting in real time and in an autonomous manner to environmental change (Ardito et al. 2019; Sanchez et al. 2020). Khan and Javaid (2022) considered that the IoT was a critical component of Industry 4.0, which improved product manufacturing efficiency, because it was done with fewer errors and costs. Somohano-Rodiríguez et al. (2022), analyzing the role that enabling digital Industry 4.0 (I4.0) technologies played in SME innovations, found that strategic planning advanced I4.0 and that enabling Information and Communication digital technologies promoted innovation more intensely than enabling digital technologies for integration and advanced robotics. Bhatia and Kumar (2022) analyzed the critical success factors of I4.0, highlighting “data governance” as the most critical factor.

The scope of the DT under Industry 4.0 expands beyond individual firms, involving the vertical integration of production systems and the horizontal integration of partners in a value chain (Tiwari 2020). Thanks to unprecedented commercial exploitation of social networks, Industry 4.0 also implies the integration of the client (Ghobakhloo 2020). Specifically, the findings of Kurniawan et al. (2022) made it clear that DT in the waste sector, not only promoted the recovery of non-biodegradable waste resources for a circular economy, but it also meant that the local community could perform online transactions of recycled goods through mobile apps. The industrial reports reveal that digitalization under Industry 4.0 offers numerous economic benefits for manufacturing, product quality, savings on operating costs, and performance in general (Dalenogare et al. 2018). Finally, if the adoption of I4.0 is a challenge for firms, the consideration of Industry 4.0 in the industry of sustainability is much more difficult (Verma et al. 2022).

## Research methodology

Bearing in mind the earlier definition of digitalization, DT, Industry 4.0, and digital technologies, this SLR of digitalization and its effects on the firm is focused on three large mature areas: management, marketing, and finance, and accounting. The main reason for this study with its SLR methodology is because it has been verified that past reviews have employed various methodologies on different digital technologies, aspects of DT, digitalization, and Industry 4.0. However, no SLR by firm area has been found that presents a picture of existing research, research gaps, and future lines of research on digitization. Therefore, the novelty of this study is (1) in going beyond another review of some aspect of digital technologies, digitization or DT; and (2) in preparing an over-arching SLR of previously published review articles -rather than empirical articles- that integrates the above-mentioned mature areas of the firm.

Its methodological approach is explained and defined in what follows. Having proposed the main goal and the RQs in the introduction, first, the identification of the data set is defined. The Web of Science database (WoS) was chosen for the literature search, because it was considered to provide both adequate and comprehensive collections of relevant academic literature (Raff et al. 2020). Second, it was decided to use PRISMA as a systematic review protocol describing the rationale, hypothesis, and planning methods of the review (Shamseer et al. 2015). The application of the PRISMA protocol has helped to create a dataset of papers. Third, content-based research was conducted to analyze the content of the papers, and to identify the main bibliographic information in each paper and the relationship with different management, marketing, finance and accounting topics. Data processing helps to identify two kinds of findings. First, the descriptive statistics that link digitalization and different topics related to management, marketing, and finance and accounting (publications by years and citations, contribution by journals, country analysis, and so on). Second, the research trends and thematic area are defined through a co-concurrence analysis of the keywords using VOSviewer software.

Co-occurrence analysis of the keywords is a technique for analyzing the primary content of selected publications (Guo et al. 2019) with which essential words may be viewed in a search field along with co-occurrences (Secinaro et al. 2022). According to Skare et al. (2022), the co-occurrence analysis of the keywords also involved an analysis of the article abstracts to identify clusters of terms. A cluster is a non-overlapping set of interlinked terms. The co-occurrence of terms in the abstracts formed various clusters. The strength of the links between terms based on co-occurrence frequency was rated.

Vosviewer software can be used to construct relations and to visualize literature based on information in the academic literature. It can display the development and the emerging publication trends of a discipline over time. VOSviewer5 software identifies predominant terms and the co-occurrence across all articles during the analysis (Biggi and Giuliani 2020). The output of the process is the clustering of terms into dominant themes.

A detailed description of the research analysis and results is provided in the following sections.

### The PRISMA protocol and the systematic literature review (SLR) approach

A Systematic Literature Review (SLR) was conducted to understand the relevant trend of studies on digitalization, adopting a consolidated approach: the PRISMA protocol (Moher et al. 2015; Nyagadza 2022). An SLR was performed to identify and to select research studies related to a specific research question for their evaluation and summary, in a fair, rigorous, and transparent manner (Chaudhary et al. 2021), so as to identify potential avenues for further research, and to highlight the boundaries of existing knowledge (Tranfield et al. 2003). Furthermore, SLR is suitable for our study, because previous research is summarized in that sort of study in a novel perspective (Chaudhary et al. 2021). Systematic reviews, as the name suggests, typically involve detailed and comprehensive planning and a search strategy designed *a priori*, so as to reduce bias (Chaudhary et al. 2021) by identifying, appraising, and summarizing all relevant studies on a particular topic (Uman 2011).

**Fig. 1:**
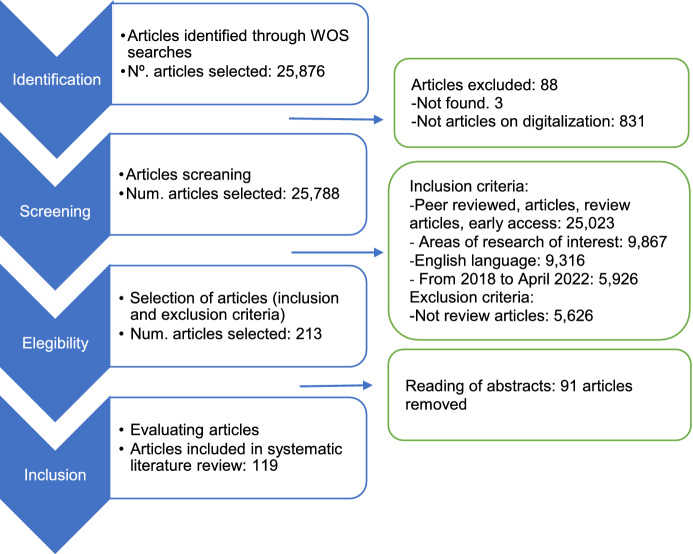
PRISMA protocol

The PRISMA protocol provides a flow diagram that helps with the steps of the SLR process: identification, screening, eligibility, and inclusion. The first purpose of this method is to plan, to identify, and to evaluate studies to extract, and to summarize data from the literature (Tranfield et al. 2003), ensuring that the bibliographic research is at once objective, transparent, and replicable. The reason for choosing this (PRISMA) protocol between other standards and guidelines (Snyder 2019) is that its analytical methodological approach is very clear and easy to understand (Moher et al. 2015). The protocol is shown below in Fig. 1.

#### Identification

The initial search on different databases showed that most relevant literature was listed in the previously mentioned WoS. In addition, the WoS Core Collection was chosen to remove the ‘noise’ that could lower the accuracy of the results. No references published in other databases such as Conference Proceedings Citation Index-Science, Emerging Sources Citation Index, and Book Citation Index-Science were considered in this study. WoS Core™ is an internationally recognized database that reflects levels of activity in scientific research and has been widely used in many studies (Thompson and Walker 2015; Yu and Liao 2016; Emmer 2018; Muhuri et al. 2019). In addition, Journal Citation Reports (JCR) is one of the most widely used publications for the evaluation of journal impact using a database of citations. Complete and neutral data are searched for in the database, which can provide a reasonable foundation for data analysis. Besides, the SSCI databases are included in WoS, making the analytic results more accurate and comprehensive (Zhou et al. 2019). The search was conducted in the WoS database on 10, 11, and 13 May, 2022.

As the purpose was to conduct a comprehensive search, in order to establish the current body of literature and knowledge in digitalization in different areas of business, management, and economics, several keywords were included in the search: digital*, “accounting”, “auditing”, “banking”, “insurance”, “taxation”, “financial structure and capital”, “marketing”, “business strategy”, “corporate governance”, “entrepreneurship”, “human resources management”, “innovation management”, “risk management”, “organization”, “production”, and “logistics”. In addition, “information systems” were included, because there might be publications within the area of business and management; and “health economics”, given its relation with the economy.

The selection of keywords and precise search strings are important and difficult tasks during the analysis of the literature (Kraus et al. 2020). The articles are identified based on the combination of keywords that could help researchers focus on the targeted literature, which are Digital* and all those topics related with Business and Management: digital* AND accounting (3,331), digital* AND auditing (332), digital* AND banking (886), digital* AND insurance (343), digital* AND taxation (71); digital* AND “financial structure and capital”(1); digital* AND marketing (5,901); digital* AND “business strategy” (193); digital* AND “corporate governance” (73), digital* AND entrepreneurship (1,232); digital* AND “human resources management” (19); digital* AND “innovation management” (84); digital* AND “risk management” (224); digital* AND organization (5,697); digital* AND production (3,535); digital* AND logistic (1,100), digital* AND “information systems” (2,771) and digital* AND “health economics” (83). These combinations of keywords were entered in the topic section of the WoS database. In each case, the number of articles appears between parentheses.

#### Screening

The following step was to revise whether each publication found after the previous step was accessible, leading to the removal of 5 articles. Then, a reading of the abstracts of the articles found under the categories “Health Economics” and Information Systems”, led to the removal of a further 83 articles.

#### Eligibility criteria

Before conducting the search, a set of inclusion and exclusion criteria were decided upon. In an preliminary phase, the inclusion criteria were publication in peer-reviewed journals, type of article, review articles and early access (25,023), documents included in the WoS were: Business, Management, Business Finance, Economics, Operations Research Management Science and Computer Science Information Systems (9,867), English language (9,316), literature from 2018-April 2022 (5,925), because an analysis of the publications from 1956-April 2022 indicated that most of them had been published between 2018 and 2021. In each case, the number of articles appears between parentheses.

Regarding the exclusion criteria, all articles that were not review articles (5,629) were removed, because the review articles were seen to predominate over the period under study. Finally, 83 duplicated articles were removed, leaving a total de 213 articles.

#### Inclusion

The next step was quality assessment including scanning the abstract and selection of papers, leaving a final sample of 119 articles (see Appendix 1). In all, 94 articles were removed for different reasons: empirical papers with a literature review, review articles that were not an SLR, and articles not directly addressing topics related to management, business, and economics.

The articles under the different categories of the WoS classification, in the areas of management, marketing, finance and accounting were grouped together to achieve the tasks described above, as may be observed in Table 1, so as to have as many articles as possible to analyze.

**Table 1:** Number of review articles under different categories of the WoS

Categories	N. review articles	Areas	N. review articles
Accounting	3	Accounting	7
Auditing	4		
Insurance	2	Finance	10
Banking	5		
Risk management	3		
Marketing	26	Marketing	26
Corporate governance	2	Management	76
Human resource management	3		
Business strategy	5		
Innovation	1		
Logistics	11		
Production	8		
Entrepreneurship	17		
Organization	29		
Total	119	Total	119

The specific questions listed in Table 2 were answered to perform the analysis of the articles.

**Table 2:** Research questions and content analysis

Questions	Specific questions	Content analysis
*RQ1*: How has the literature on digitalization developed up until 2022 in relation to different areas of the firm: management, marketing, and finance and accounting?	*RQ1.1.* Which articles are crucial in the development of digitalization?	*Calculating publications and citations*
	RQ1.2 What are the production levels and the impacts of authors, journals, and research areas?	*Calculating the ranks*
RQ2: What are the principal research topics in relation to digitalization within the different areas of the firm: management, marketing, and finance and accounting?	Q2.1 What are the major research subjects concerning digitalization?	*Cluster analysis*
RQ3: What is the research agenda in relation to digitalization within the different areas of the firm: management, marketing, and finance and accounting?	RQ3. What are the major research gaps concerning digitalization?	

## Results

### How has the literature on digitalization developed up until 2022 in relation to different areas of the firm: marketing, management, and finance and accounting?

In this section, the main analyses of the corpus are presented, as described in Sect. 3, starting with the distribution of the papers on a yearly basis, the scientific journal of publication, and the country. These analyses are performed, in order to understand how the interest in digitalization related to business economics has been gaining importance over time.

**Fig. 2:**
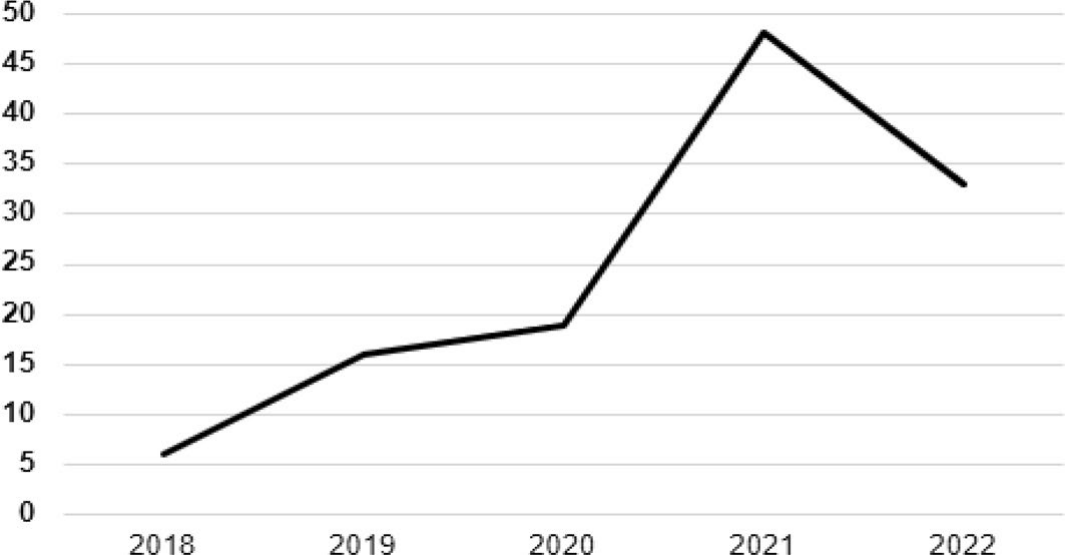
Number of articles by year (2018–2022)

In Fig. 2, a substantial increase of review articles may be observed as from 2020, whereas before that year, the number of publications was less frequent. This item of information was the principal reason for this SLR on review articles and not on articles. An analysis of the periods of study of the review articles supports the earlier results, because 27.87% of the review articles under analysis were undertaken within a time frame up until 2020, 21.31% within a time frame up until 2019, and 15.57% of the SLR, up until 2021.

**Fig. 3:**
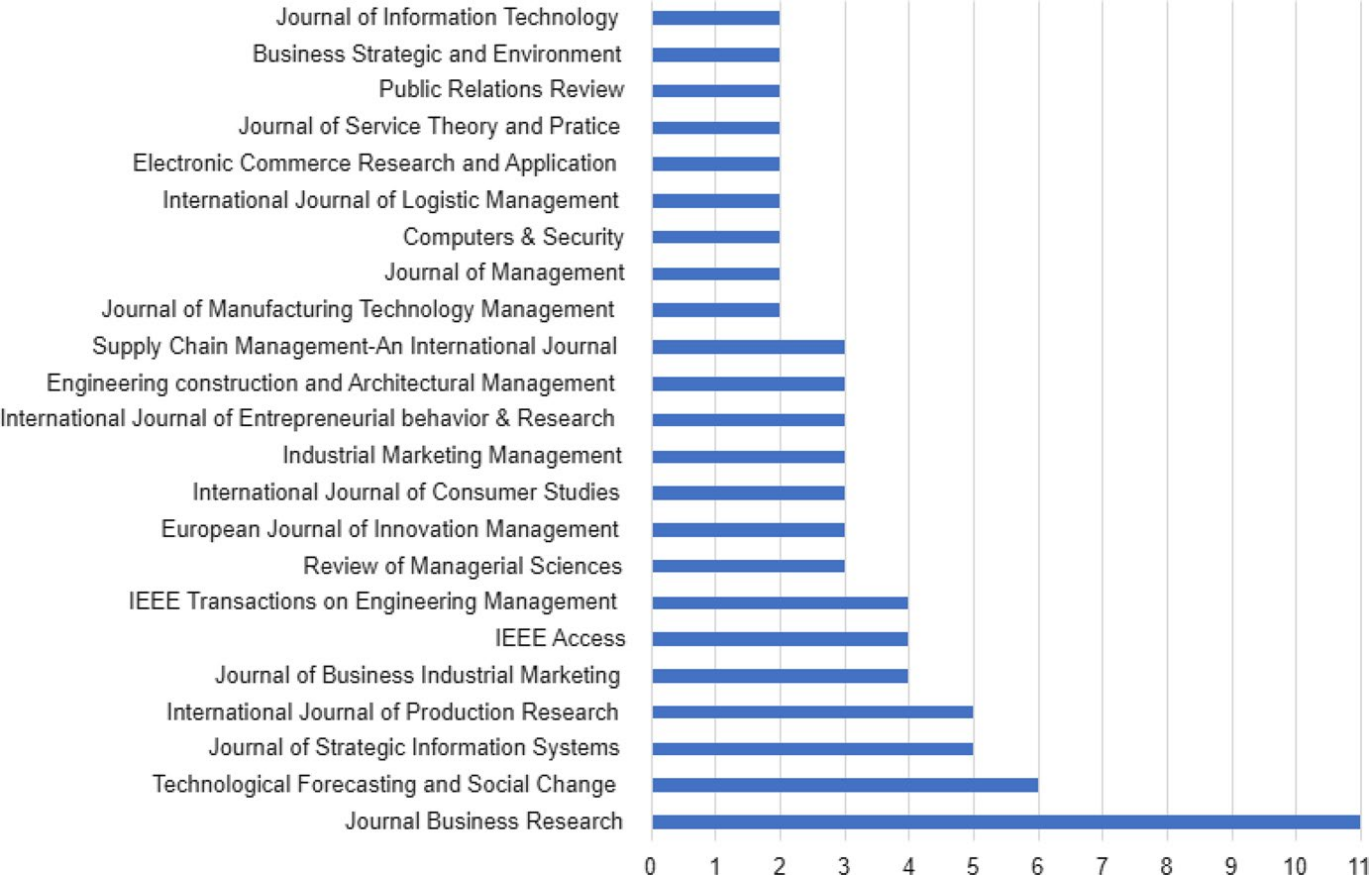
Number of articles by publication (2018–2022)

Although the corpus included articles from 76 different journals, 50.82% of the papers in the corpus were published in 50 journals and another 49.18% were published in 26 journals, reflecting high fragmentation within the literature. Figure 3 reports the journals that have published at least two papers on the topics of our study. The fragmentation observed in the literature is owing to 31 journals that are closely linked to the disciplines of accounting and finance, management, business and marketing, while the others are related with transversal and multi-disciplinary issues. Thus, some papers of the corpus have been published in journals that are focused more on operations (*i*.*e*., Annals of Operations Research), transportation (*i*.*e*., Research on Transportation Economics), and sectors (Engineering construction and Architectural Management).

**Fig. 4:**
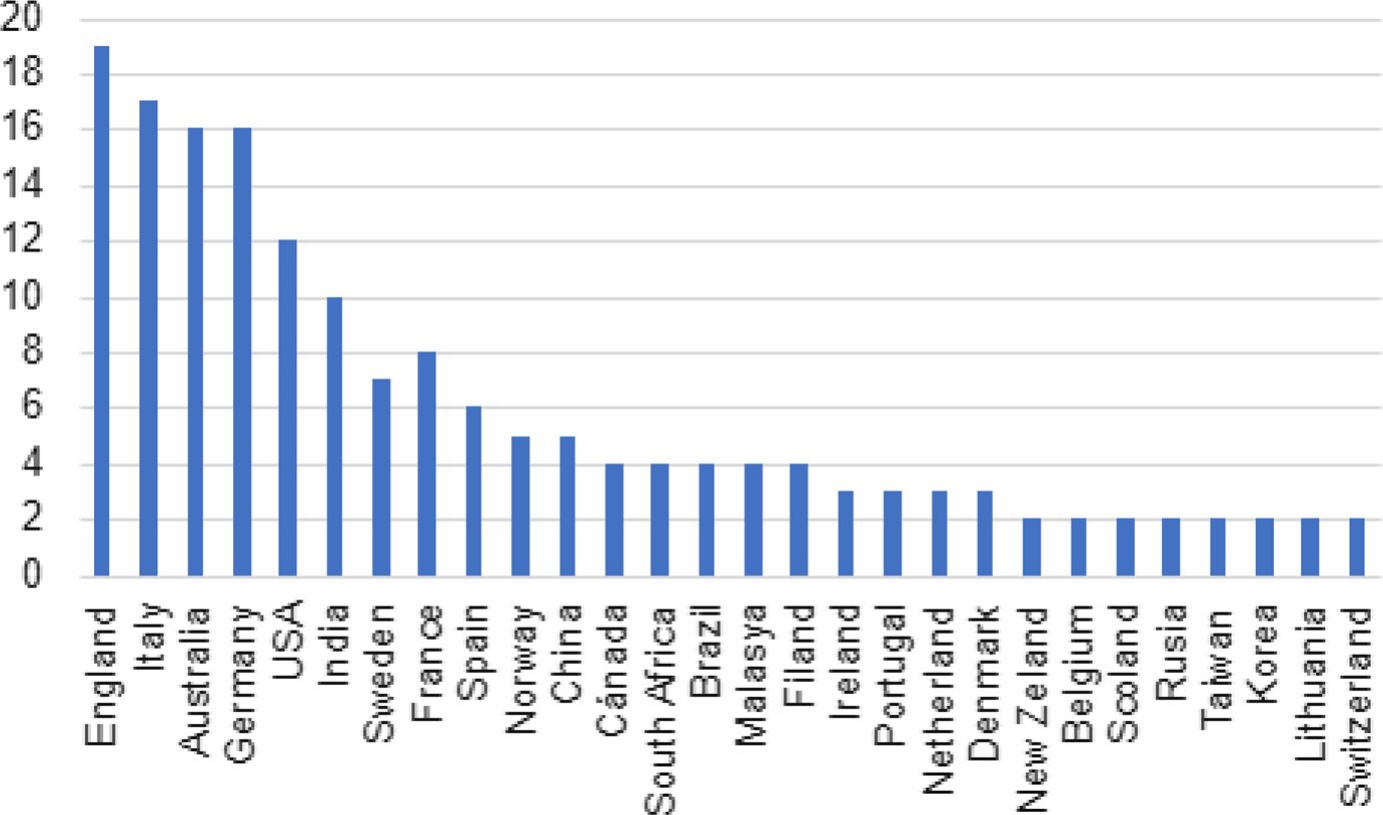
Number of articles by country (2018–2022)

In Fig. 4, it may be seen that Europe leads the number of published review articles with almost 50% of the publications shared between England, Italy, Germany, and France. The Australian continent follows on with 14.75% between Australia and New Zealand, and finally North America with 13.12% between the USA and Canada. 8.20% of the corpus under analysis was published in India.

The references from each of the 119 articles under consideration in this study were analyzed to respond to questions RQ1.1 and RQ1.2. In addition, the references of each of the 119 articles with a number of citations ≥ 100 were selected, as the purpose was to highlight those articles that have been cited most of all among the articles under analysis and the most influential articles. A total of 764 references with 100 or more citations were extracted from the 26 articles on marketing, of which only 8.25% had been cited two or more times in the articles under study.

**Fig. 5:**
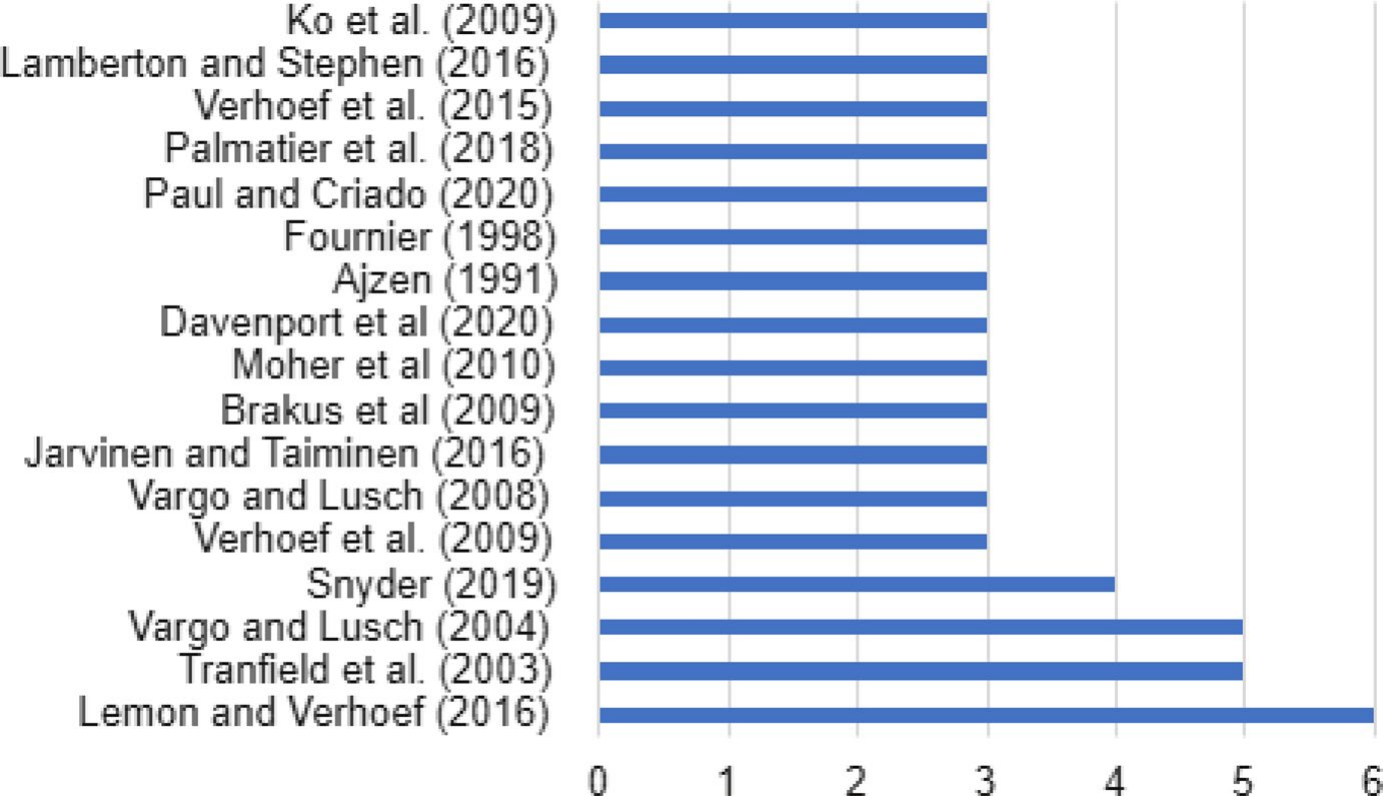
Most frequently cited authors of review article published in the field of marketing and digitalization

In Fig. 5, the most frequently cited marketing publications may be seen, highlighting the article of Lemon, K. and Verhoerf, P., titled *Understanding customer experience throughout the customer journey* published in 2016 in the *Journal of Marketing*. However, the most influential article with a total of 3,670 citations (367 citations per year) was published in 2012 by the authors Venkatesh, V., Tong, J and Xu, X in *MIS Quarterly* titled *Consumer acceptance and use of information technology: Extending the unified theory of acceptance and use of technology.* In Table 3, other articles may be seen that were also influential in the field of marketing and digitalization.

It may be seen that Peter Verhoef is one of the most cited authors within the field of digitalization and marketing. He has three articles among the most frequently cited in the articles under analysis each of which has a high number of citations forming a network with Katherine Lemmon.

**Table 3:** Top 10 Marketing references with the highest average number of citations per year

Year	References	Citations among review articles	Citations	Average citations per year
2012	Venkatesh et al.	2	3,670	367.00
2003	Tranfield et al.	5	4,518	237.79
2008	Vargo and Lusch	3	3,317	236.93
2016	Lemon and Verhoef	6	1,251	208.50
2002	Webster and Watson	2	2,733	136.65
2020	Davenport et al.	3	249	124.50
2015	Verhoef et al.	3	865	123.57
2020	Paul and Criado	3	247	123.50
2015	Ostrom et al.	2	740	105.71
2009	Verhoef et al.	3	1,190	91.54

**Fig. 6:**
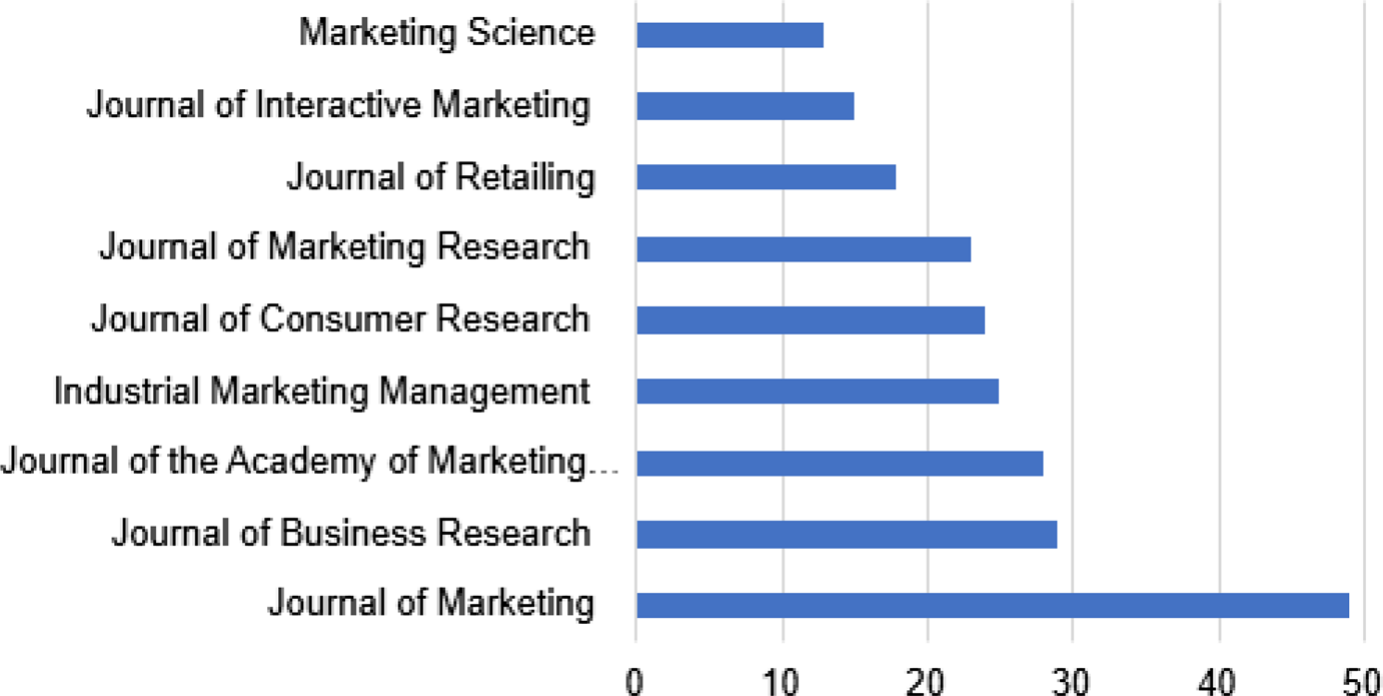
Top 10 most productive sources

In Fig. 6, the most productive source in marketing may be seen in the field of digitalization. All journals are specialized in marketing except the Journal of Business Research that is more generalist.

Of the 79 articles on management, 2,606 references with 100 or more citations were found, of which 18% were cited at least twice in the articles under analysis.

**Fig. 7:**
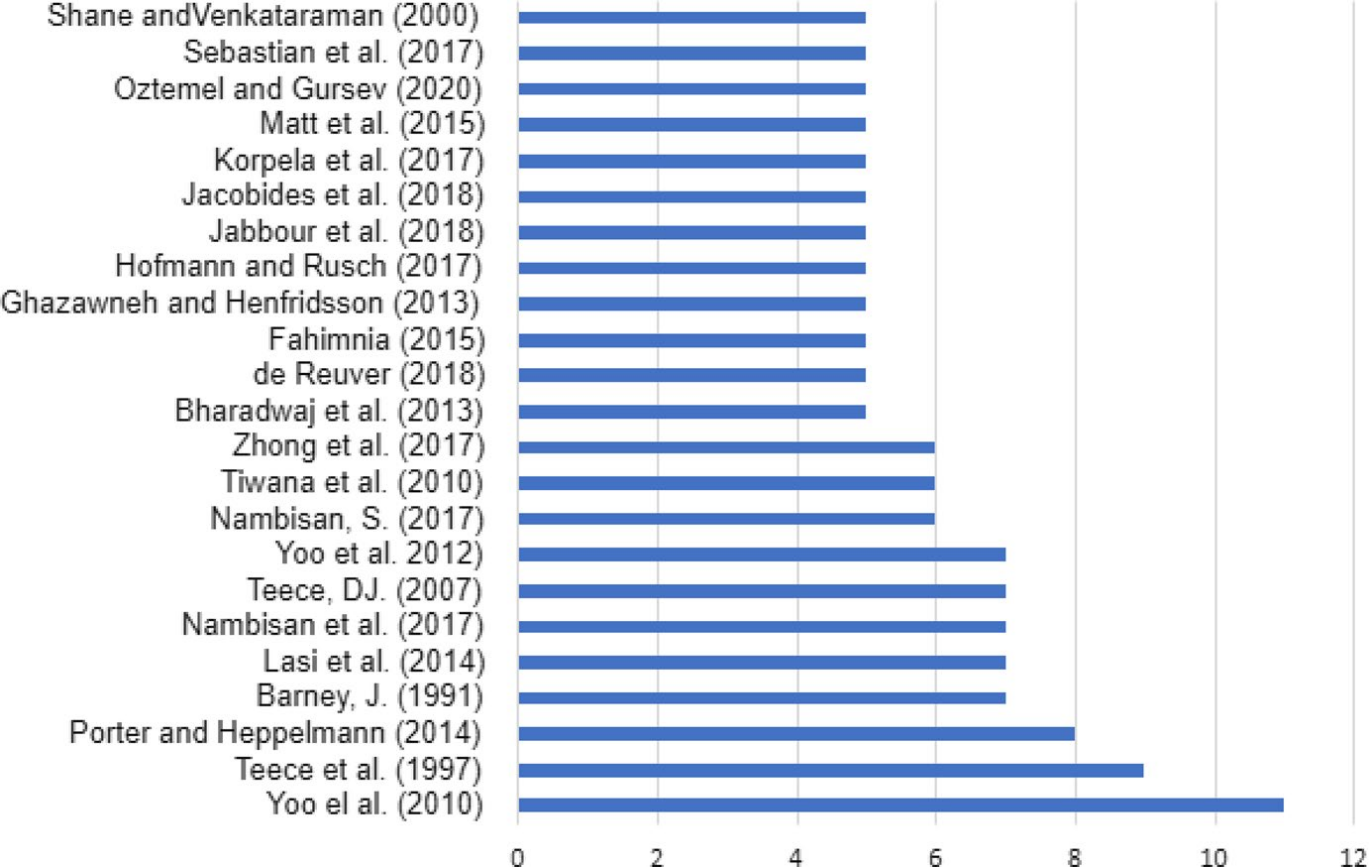
Most frequently cited authors of review articles published in the field of management and digitalization

In Fig. 7, the most frequently cited articles with at least 5 citations from the corpus may be seen. The article that stands out with 9% of all citations is (11) *“The New Organizing Logic of Digital Innovation: an Agenda for Information Systems Research”* by the authors Yoo, Y. J. Henfridsson, O. and Lyytinen, K., written in 2010, and published in *Information Systems Research*. These authors stand out for having created a research network on organization and digitalization.

In Table 4, the most influential articles in the field of digitalization and management can be seen. With almost 754 citations, Barney, J. (1991) is the most influential author with his article *Firm resources and sustained competitive advantage* published in the *Journal of Management*. Nevertheless, it is also worth mentioning the article titled *Literature review of industry 4.0 and related technologies* written by E. Oztemet, and S. Gursev (2020) in the *Journal of Intelligent Manufacturing* with 218 citations by year.

**Table 4:** Top 10 management-related references with the highest average number of citations per year

Year	References	Citations among review articles	Citations	Average citations per year
1991	Barney	7	23,365	753.71
1997	Teece et al.	9	13,822	552.88
2007	Teece	7	5,022	334.80
2000	Sanhe and Venkataraman	5	5,772	262.32
2020	Oztemet and Gursev	5	436	218.00
2014	Lasi et al.	7	1,599	199.86
2017	Zhong et al.	6	855	171.00
2018	Jacobides et al.	5	629	157.25
2014	Porter and Heppelmann	8	1,004	125.50
2017	Hofmann and Rusch	5	625	125.00

**Fig. 8:**
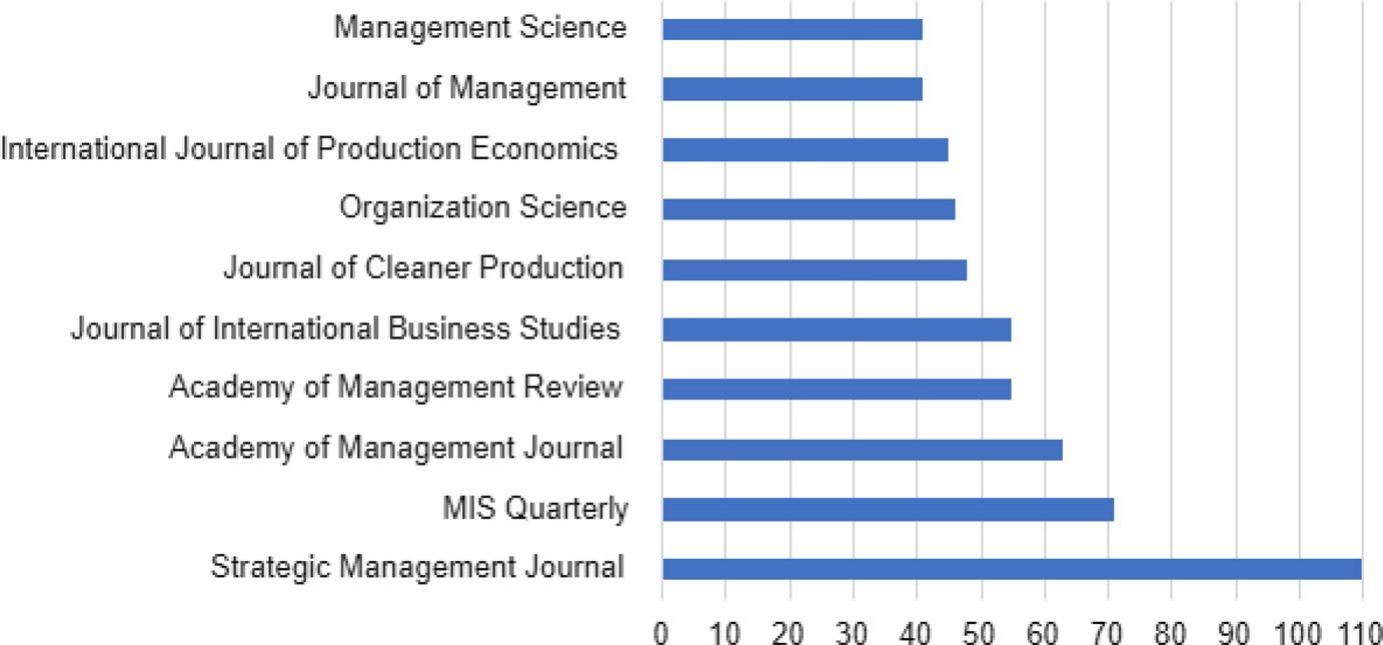
Top 10 most productive sources

In Fig. 8, the most productive source of management in the field of digitalization can be seen. Most journals are within the field of management. The *Strategic Management Journal* stands out amongst the others as the journal that has published most of all on digitalization and management.

Finally, of the 17 articles on finance and accounting, 100 of the 543 references were cited, of which 17.31% were cited at least twice in the articles analyzed in this area.

**Fig. 9:**
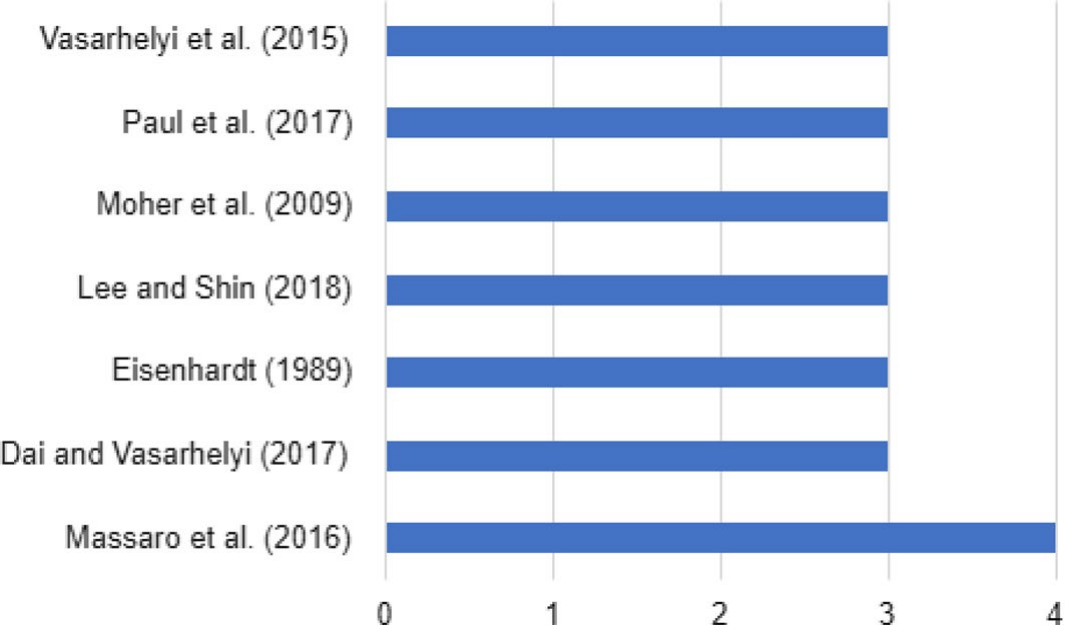
Most frequently cited authors of review articles published in the field of finance-accounting and digitalization

In Fig. 9, the most widely cited publications can be seen in digitalization, and finance and accounting, highlighting the work of the Massaro, M., Dumay, J., and Guthrie, J. (2016) titled *“On The Shoulders of Giants: Undertaking a Structured Literature Review in Accounting”* in *Accounting, Auditing & Accountability Journal*.

Among the most widely cited references, the most influential is one from the authors Gossling, S., Scott, D., and Hall, C.M. (2021) titled *Pandemics, Tourism and Global Change: a Rapid Assessment of Covid-19*, published in the *Journal of Sustainable Tourism* with a total of 1,187 citations (1,187 in one year). This article is within risk management in the area of finance. In Table 5, it may be seen that there are other references that even though less cited among the corpus of finance and accounting under analysis have a larger number of citations. No cooperative network with a higher number of citations has been observed. No cooperative research network was observed among the authors under analysis.

**Table 5:** Top 10 finance-accounting and digitalization-related references with the highest average number of citations per year

Year	References	Citations among review articles	Citations	Average citations per year
2021	Gossling et al.	2	1,187	1,187
2021	Wen et al.	2	241	241
2014	Mollick	2	1,546	193.25
2020	Hall et al.	2	345	172.5
2021	Neuburger and Egger	2	144	144
2021	Kaushal and Srivastava	2	141	141
2021	Zheng et al.	2	139	139
2020	Higgins and Desbiolles	2	274	137
2020	Zenker and Kock	2	260	130
2020	Jiang and Wen	2	234	117

**Fig. 10:**
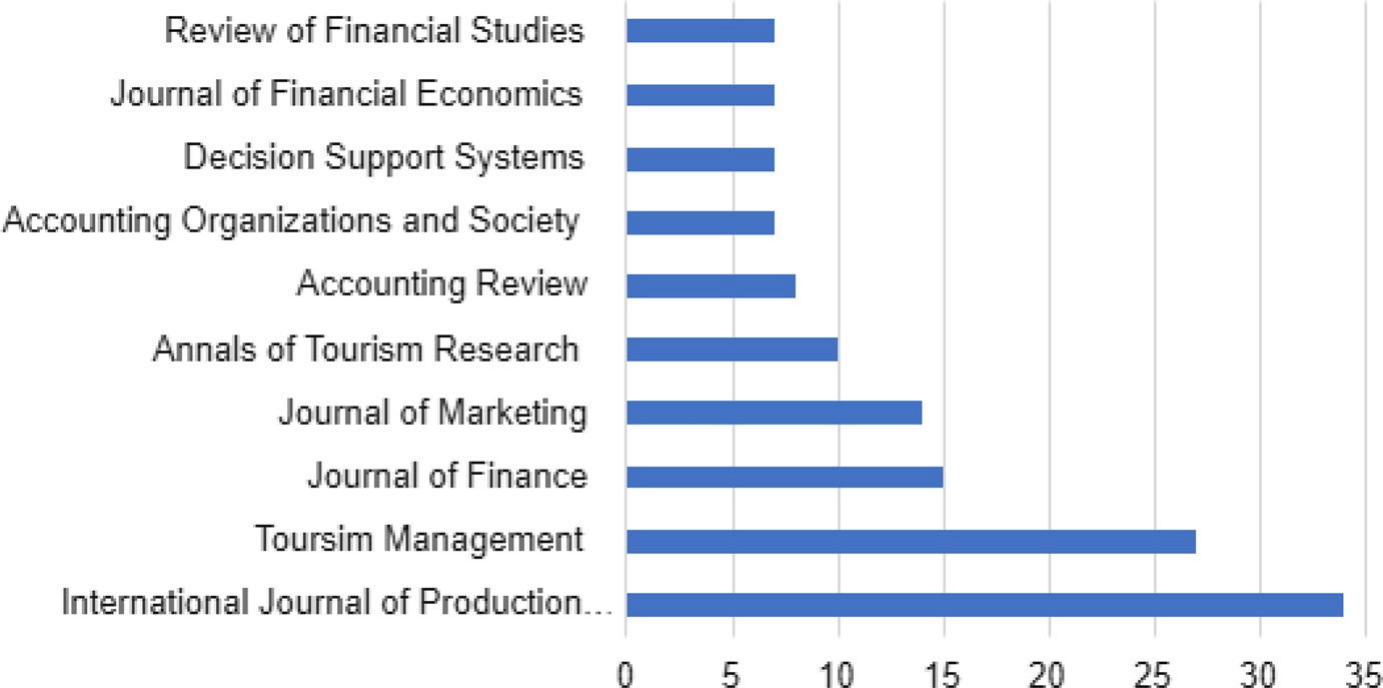
Top 10 most productive sources

In Fig. 10, the most productive sources in finance and accounting in the field of digitalization can be observed. Most of the journals are not within the field of finance and accounting. The *Journal of Finance* stands out as the specialized journal in which most papers on digitalization and finance and accounting have been published.

### Principal topics and analysis of research trends within marketing, management, and finance and accounting

A co-occurrence analysis was completed to respond to RQ2 and to establish the topics of interest of both the set of articles on digitalization and each functional area of the firm: accounting and finances, marketing, and management. The analysis was performed in two ways, to obtain results of greater reliability: based on keywords and based on titles and abstracts. Both gave the same results for the co-occurrence analysis in the 3 areas under analysis. The analysis based on keywords, titles, and abstracts is therefore described below.

First, the groups formed on the basis of titles, abstracts, and keywords were sought in the set of articles for their identification (VanEck and Waltman 2010). VOSviewer 1.6.15 (VanEck and Waltman 2010) was employed for this analysis. A minimum number of five occurrences to consider a term is suggested for this tool. However, in this analysis, the number of occurrences was varied as a function of the number of articles that were analyzed. The generic terms such as “article”, “review”, “systematic review”, “systematic literature review” were removed during the data cleaning process, as it was a review of a sample of articles. In addition, similar terms were grouped under one single term (van Eck and Wallman 2010, 2020), such as “Covid-19”, “Covid” and “pandemic”, or B2B and “business-to-business”. In the following figures, the clusters obtained for each area are shown. The nodes represent keywords or concepts, while their size reflects their frequency (van Eck and Waltman 2010, 2020). VOSviewer represented each group of keywords with a different color. This analysis was repeated within each and every area.

#### Marketing

**Fig. 11:**
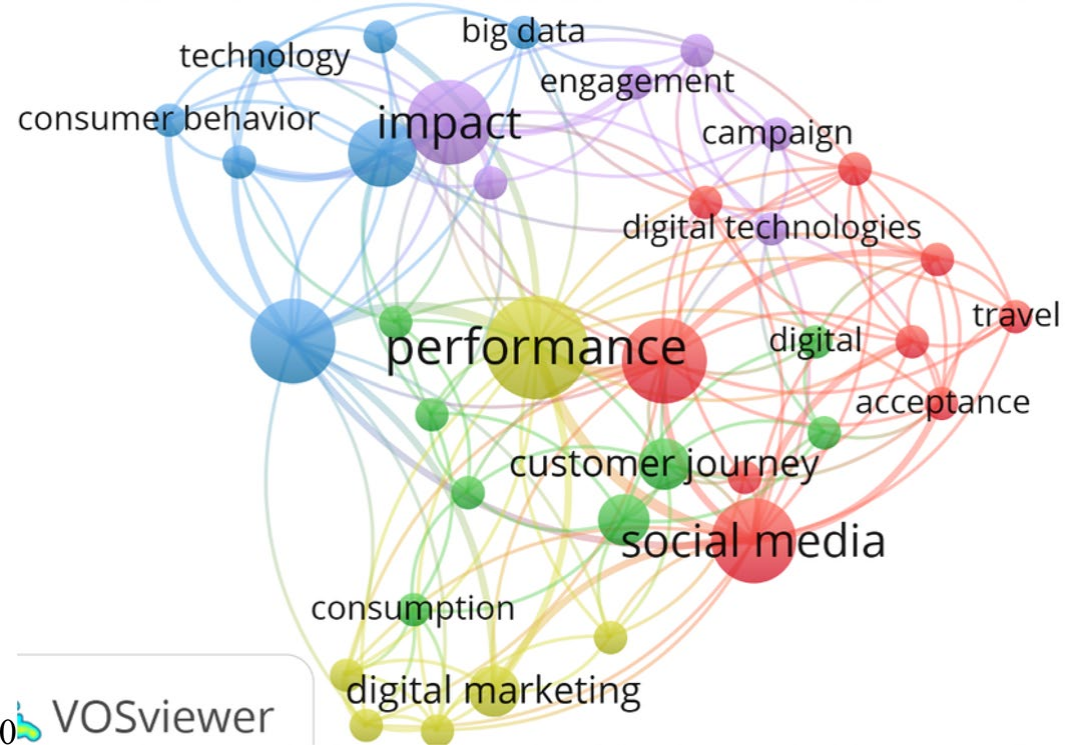
The result of the co-occurrence analysis in the field of marketing and digitalization

In the area of marketing, we set a minimum of 2 occurrences for the analysis of concurrence, for a term to be considered in the programme, because we had a relatively low number of (26) publications. The most frequency cited topics gave us an idea of the structure of the topics that have been investigated most of all and that have contributed to the development of digitalization in marketing. In Fig. 11, the 5 clusters obtained from the previously detailed co-occurrence analysis are shown.

- Cluster 1 has “performance” as a highlighted node and groups other keywords such as “digital marketing”, “co-creation”, “customer engagement”, “adoption”, “customers”. Thus, the cluster is related with digital marketing performance and is centered on adoption and engagement from the perspective of the consumer.
- Cluster 2 has “social media” as a highlighted node and groups together other keywords such as “management”, “digital technologies”, “travel”, purchase intention”, “acceptance”, “Word-of-Mouth” (WOM), and “strategy”. This cluster is related with the acceptance and management of social media as a marketing strategy, directed at WOM and purchase intention of the consumer. The most frequently cited and therefore the most investigated topics were WOM, purchase intention, and digital technologies.
- Cluster 3 has “knowledge” as a highlighted node and groups other keywords such as “big data”, “consumer behavior”, “Covid 19”, “business”, and “decision-making”. This cluster was related with knowledge of consumer behavior through big-data technology, above all during the Covid-19 pandemic, to facilitate decision-taking within firms.
- Cluster 4 has “impact” as the highlighted node and groups other keywords such as “relationship management”, “engagement”, “campaign”, and “digital technologies”. This cluster is related with the impact of mass-media campaigns through digital technologies measured through engagement and managerial relations. Most present-day studies have examined the acquisition of a mobile device in the context of publicity and the marketing of services.

Finally, Cluster 5 has “customer journey” as a highlighted node and it groups together other key words such as “customer experience”, “online”, “innovation”, and “dominant logic”. This cluster is related with the customer journey via online outlets for the purchase of a product and the product experience.

In short, social networks have been turned into the most influential channel of digital marketing, creating an imminent need for the specialists in digital marketing to exploit transformative marketing even more so. Compiling data from social networks for the analysis of consumer opinion (Micu et al. 2018) has created additional opportunities to explore programs created on social media platforms to compile, to analyze, and to understand consumer data.

#### Management

**Fig. 12:**
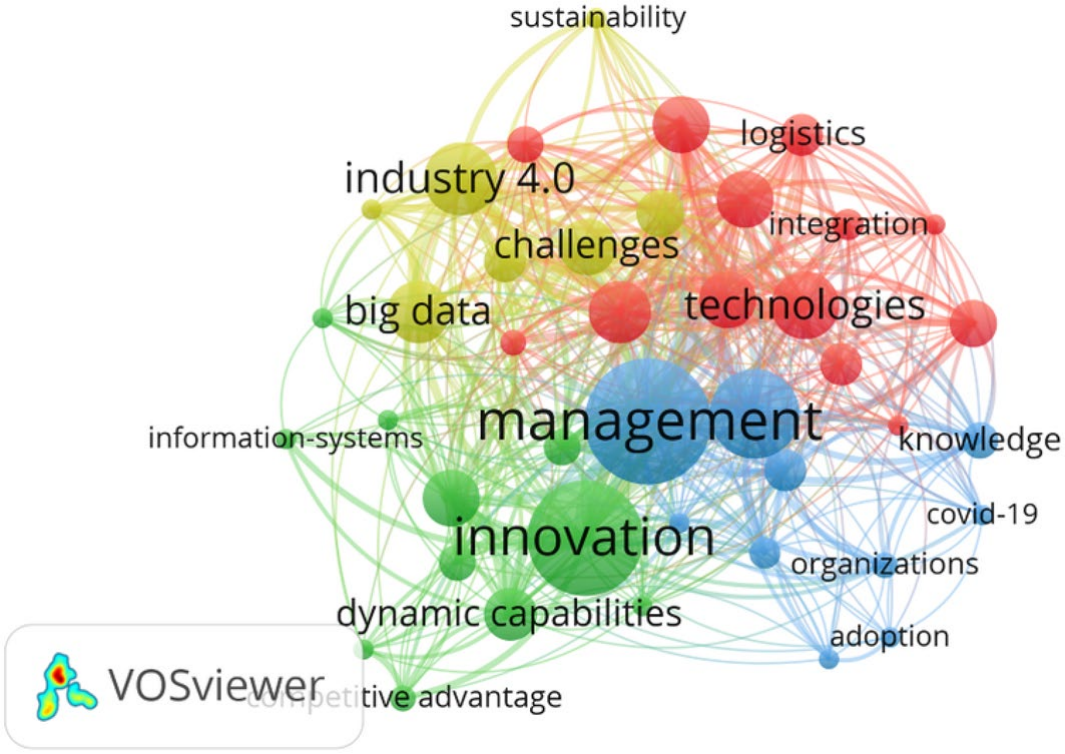
The result of the co-occurrence analysis of review articles in the field of management and digitalization

We had 76 publications within the area of management to conduct the co-occurrence analysis. As it is a greater number of articles than in the case of marketing, the terms were required to appear in at least 4 publications. The 4 clusters that were obtained may be observed in Fig. 12:

- Cluster 1 has “management” as a highlighted node and groups together other keywords such as: “performance”, “information”, “knowledge” “business”, “organizations”, “communities”, and “Covid-19”. This cluster is related with the management and the performance of information to convert it into knowledge for organizations, businesses, and communities.
- Cluster 2 has “innovation” as a highlighted node and groups together other keywords such as: “digital transformation”, “dynamic capabilities”, “information technology”, “big data analytics”, information systems”, “value creation”, “firm performance”, “competitive advantage” and cyber-physics. This cluster is related with innovation in everything related with DT that affects dynamic capabilities, information technology, information systems, value creation, competitive advantage, and firm performance. Briefly, it could be said that it is related with malleable organizational designs (Hanelt et al. 2021), so that firms that are easily influenceable and flexible can continue adapting to their environments.
- Cluster 3 has “technologies” as the highlighted node and groups together other keywords such as “internet”, “supply-chain management”, “impact”, “logistics”, “entrepreneurship”, “business model”, and digitalization”. This cluster is related with Internet technologies and digitalization that impact on logistics, and on supply chain management, as well as those that set up business models and favor entrepreneurship. It could be said to be related with the ecosystems of digital business models (Hanelt et al. 2021).
- Cluster 4 has “Industry 4.0” as the highlighted node and groups together keywords such as “big data”, “challenges”, and “future”. This cluster is related with big data and its appearance in the firm, as a future challenge.

In short, the closeness between the nodes indicates that all the topics are interrelated, even though they belong to different clusters. Sustainability may be highlighted as the most distant topic.

#### Finance and accounting

**Fig. 13:**
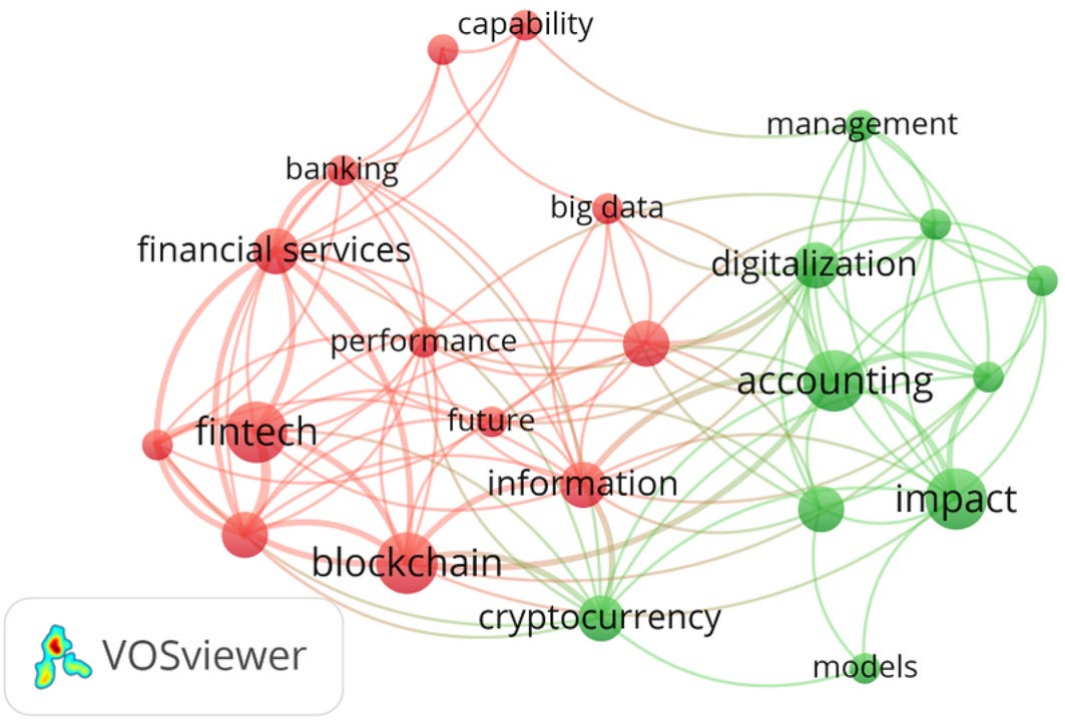
The result of the co-occurrence analysis in the field of finance-accounting and digitalization

The areas of Finance and Accounting were linked, because the number of articles in each one of them was excessively small to conduct the analysis. In this way, there were 17 articles to complete the co-occurrence analysis. The minimum chosen number of occurrences was 2. Two clusters appear in Fig. 13.

“Fintech” and “Blockchain” were the most highlighted nodes within Cluster 1 and grouped together such words as “financial technologies”, “innovation”, “information”, “financial services”, and banking”. This cluster was related with technologies such as blockchain applied to financial services (Fintech) and to banking. It may be said that mainstream commercial banks accept the impact of technology, the way in which financial services are changing, and the way new business models are gaining ground in the financial industry. A large number of “Fintech” firms, “Fintech” industries, and “Fintech” markets have emerged.

The most highlighted node in Cluster 2, “accounting”, grouped together such keywords as “impact”, “cryptocurrency” “digitalization”, “risk management”. This cluster is related with the digitalization of accounting, the impact of cryptocurrency, and risk management. Cryptocurrency is within a different cluster to the one in which blockchain belongs, but it is close enough, because cryptocurrency cannot be analyzed without mentioning the technological foundation of cryptocurrency: blockchain technology.

## Findings

In this section, the findings of this SLR conducted within the field of digitalization in business economics are reported.

### Marketing

The progress of marketing throughout this period where digitalization has dominated can be summarized in the following way. When consumers identified search engines as a means of searching for information, studies on marketing through search engines emerged (Dou et al. 2001), giving rise to a new area of investigation: digital marketing. In turn, the studies within this area accelerated the growth of the “e-WOM” or electronic Word-of-Mouth, between 2000 and 2004. Two articles by Dellarocas (2003), and Godes and Mayzlin (2004) may be highlighted. The latter author discovered the interaction between offline and online environments, giving rise to three concepts that were to be the source of research over the coming decade: Zero-Moment-Of-Truth (ZMOT) (Lecinski 2011), webrooming (Flavian et al. 2016), and single-channel retailing (Blom et al. 2017).

In the retail trade, as the level of consumer engagement with online retailing formats has progressed, the focal interest of research has shifted towards the online experiences (Novak et al. 2000) and their value and costs. The quality of the virtual experience with electronic services has led researchers to investigate the concept of the quality of the electronic service, analyzed from the point of view of the client (Yi and Gong 2008), the system (Bronner and Kuijlen 2007), and the retailer (Venkatesan et al. 2007). Consumer privacy has been turned into one of the central research topics, with the compilation, storage, and distribution of consumer data (Norberg et al. 2007). With the growth of single-channel retailing, studies on consumer responses to the delivery of value went beyond decision making. Retail commerce understood as an extension of brand communities, investigative attention has been centered on brand attachment, love and hate, and different emotional responses of consumers to marketing stimuli (Bleier et al. 2019; Petit et al. 2019).

The above-mentioned lines of investigation have led researchers to center their attention on the concept of value or the delivery of value to facilitate persuasion, and consumer satisfaction and loyalty. Analyzing the concept of value and its meaning for the consumer in the digital environment (Gallarza et al. 2011), taking a step forward, marketing research began to analyze another two value components: utilitarian and hedonic values (Childers et al. 2001).

Digital advertising emerges and more specifically, the effectiveness of publicity, measured through clickstream data that has become a new line of investigation (Tucker 2014). The rapid improvements to digital publicity gave rise to new techniques such as retargeting through online offers (Berger and Milkman 2012), and other topics such as user-generated content, online reviews (Floyd et al. 2014), online offers, and WOM marketing (King et al. 2014).

Up until 2014, topics such as multi-channels and mobiles remained emergent yet unexplored investigative areas (Lamberton and Stephen 2016). It was after the article of de Verhoef et al. (2015) when single-channel marketing captured growing interest in the investigation (with early examples such as Yoo and Lee 2011; Dinner et al. 2014;). With regard to mobile telephony, Atkinson (2013) produced the earliest study on the use of QR codes for purchases. As consumers gained greater familiarity with digital technologies and started to interact with digital channels in different ways, investigations began into the analysis of their emotional responses to marketing, and into consumer commitment towards digital channels and digital mediums. A new tool appeared for the marketing specialists: “User-Generated Content” (UGC) as an extension of eWOM that had been one of the previously analyzed topics. The works on UGC were gradually diversified towards online review (Bilro et al. 2019; Van Laer et al. 2019), eWOM (Chu and Kim 2018), and the content generated in online brand communities (Hollebeek et al. 2017).

In the field of consumers, the investigation has been centered on such topics as consumer participation in social networks and the co-creation of value. Particularly, collaborative consumption and social exchanges have been present since the first decade of the new Millennium, with the first investigations on renting loans and the exchange of different articles using digital platforms (Mohlmann 2015; Hwang and Griffiths 2017; Dellaert 2019). As digital forums are consolidated, digital products are also created such as social network accounts, games, music, videos, and avatars, giving rise to a new investigative topic: digital music and the psychological property of digital goods among consumers (Bardhi and Eckhardt 2017). This topic is an extension of the virtual experiences analyzed earlier.

### Management

It is difficult to describe the progress of research over time into digitalization in the field of management, because consideration must be given to each aspect of the organization of a firm: supply chain management, logistics, manufacturing, human resources management, knowledge management, business management, entrepreneurship. We have therefore centered on the more general lines of research.

The firm has to have the capability to detect and to take advantage of interruptions, through strategic responses, and to reorganize its business model components, when faced with DT. Therefore, dynamic capabilities are a topic of investigation, which has been centered on: (1) adapting these capabilities to support DT; (2) integrating the capabilities into the reality of digital platforms and ecosystems; and (3) sustaining them through a micro-analysis from the perspective of the Chief Digital Officer.

One pattern that is observed in the DT are the malleable organizational designs based on digital technologies and agile structures for rapid adaptation to environmental opportunities and threats. Data-based operations and decision making are upheld by automated virtual commercial processes, as well as technology-centered management. Finally, the results of the digital business models centered on client and digital experience, and intelligent connected and personalized products can be related with the mechanism of instantaneous launches, because it is a matter of dynamically adaptable and scalable market offers.

In the context of a DT, it is easy for firms to become aware of the importance of malleable organizational design. Both access to large volumes of data and the technologies for their analysis, for detecting changes within the business environment and the capability to adapt activities that are based on flexible digital technologies prompt continuous changes on the basis of environmental feedback, and emerging opportunities. At the same time, however, it is more difficult for the firm to distinguish the source of the change and if its development is within or beyond its limits, because it may be provoked by external agents such as clients and developers (Parker et al. 2017). In addition, a large part of the technological configuration might be beyond the control of a given firm and might be developed in an unpredictable way, under the will of external actors, such as technological giants and new digital firms. Operations and decisions based on data adjust themselves to the commentaries within a business setting, for example from the clients, when using the potential of Artificial Intelligence (AI) and automatic learning to detect changes and to react automatically to them. Rapid transformation requires firms to modify their stance and capabilities for agreement with the new opportunities that dynamically emerge and die down. Therefore, in the course of the DT, continual adaptation assisted by malleable organizational designs contribute to a wholistic confluence of the turbulence within business settings, information technological systems, and organizational capabilities. A situation known as “digital ecodynamics”, which “has no separations between its three central elements, as it is rather the fusion of all the interactions between all three elements” (El Sawy et al. 2010, p. 837).

The appearance of digital business ecosystems is another topic of DT within the firm. These ecosystems define business environments “formed by a network of interdependencies specifically generated through digital technologies” (Kopalle et al. 2020, pp. 114–15). As some shape is given to the decisions of the firm on where and how to compete, business ecosystems are an essential topic for the strategy (Jacobides et al. 2018). Over past decades, numerous firms have distanced themselves from integrated and hierarchized supply chains and are moving closer to more fragmented networks of strategic associations with external entities (Bitran et al. 2007). However, over the course of a DT, the digital business ecosystems are now not only interesting for information technology and software industries, but they are also increasingly relevant in all sectors as digital technologies spread throughout industry and society.

A key differentiating factor that distinguishes digital business ecosystems is their turbulent nature (El Sawy and Perreira 2013). In the context of entrepreneurial ecosystems, this turbulence is especially visible in the great quantity and heterogeneity of interdependent partners who give shape to the competition (Jacobides et al. 2018), the generalized diffusion and the adoption of generative, interconnected technologies (Kopalle et al. 2020), and the preferences of clients that undergo constant change (Downes and Nunes 2013). The research emphasizes the relevance of these factors and, therefore, a general movement towards digital business ecosystems (Bouncken and Kraus 2022). For example, it is evident that DT is activated and molded by a large quantity of different digital technologies and applications such as blockchain technologies, IA, and IoTs. In addition, consumers increasingly incorporate technology into their daily routines and personal interactions, which has profound implications for their demands and expectations with respect to the offers and the communications with firms.

Firms must now compete with a larger number of competitors, numerous industries and, in some cases, with completely different commercial models. A change may be appreciated towards interconnected markets in which the participants are involved in numerous networks of exchange that grow and dissolve very easily. This sort of change can mainly be attributed to the ubiquity of Internet and other related technologies upon which these networks in large measure depend, which in general leads to digitally permeated markets. In addition, the convergence of previously separated industries may be observed as heterogenous actors from different industries increasingly operate and compete within the same markets, due to the possibilities of digital technologies, which amass the experiences of previously disconnected users (*i*.*e*., Broad-band Internet, telephony, and television) (Yoo et al. 2012).

Due to the growing turbulence, digital business ecosystems are “changing the rules of the game in many industries through the disruption of business models” (Pagani 2013 p. 617). However, among the digital business ecosystems, the value proposals that are followed can radically change within a short period of time (Yoo et al. 2012). The participants within the digital business ecosystems, their positions, and their roles are subject to constant changes. In addition, firms co-create and co-capture value with a great variety of heterogenous actors who go beyond suppliers and traditional clients and range from individuals to communities of emergent firms, technological giants, and social actors (Rubio-Andrés et al. 2022; El Sawy and Perreira 2013). A completely new level of interconnections and interdependency arises and undergoes constant growth, due to the low entry barriers for practically everybody.

In summary, the change towards a flexible organizational design is integrated and is driven by the digital business ecosystems and cannot be considered in isolation (Hanelt et al. 2021). This relation is affected by the specific aspects and changes related with the conditions of the business environment. The scope of the environment can vary throughout one continuum, from a “narrow” scope, which is generally focused on specific elements related to the digital business ecosystems and their relation with organizational design, up to a “broad” scope examining thematic patterns of the DT in a more holistic way.

### Finance and accounting

Financial technology appeared for the first time in 2000, which suggests that the technology might have been the basis for the development of the Fintech industry. While some researchers are exploring how banks can use strategies related with entrepreneurial orientation to achieve an excellent performance in the digital era (Niemand et al. 2021), others are investigating how fully virtual operations affect the experience of service and financial habits of users (Windasari et al. 2022). In addition, the demand for money and the financial markets are not only salient topics of investigation of Fintech, but they are also classic topics when investigating the financial industry.

In a period of rapid development of Fintech, many hot terms were generated between 2016 and 2018 such as the regulation of digital currencies and banking sectors, financial transactions and their success, the Fintech industry in global banks, and the new Fintech firms. In-depth AI learning, blockchain, and other information technologies were also investigated (Marinakis and White 2022), as well as the digital wallet, mobile payment service models, P2P lending, and equity crowdfunding transaction models.

The new topics that emerged between 2018 and 2021 reflected the most recent Fintech-related developments. First, the traditional banking industry occupies an essential position within traditional finance, and the banking system and the digital bank are the last directions for development to obtain competitive advantage. Second, the intelligent contract and the payment system is a new and important technology for digital finance. Third, an increasing number of studies have centered on the reputational scores of individual investors and consumer satisfaction. Competition between banks and the efficiency of the processes are therefore important factors that test the development of firms that finance technology. Fourth, crowdfunding and blockchain are two different forms of altering and innovating traditional financial intermediaries, achieving the transformation of a business model (Cai 2018).

Information technology provides guarantees for problems of consumer security. The topic of smart data storage to protect both the privacy and the trust of the consumer is principally related with the field of information safety, which is supported by information-privacy technology, and encryption technology. Academics have launched a series of studies on data-security questions: smart data storage protected by privacy for the financial industry through cloud-computing technology (Qiu et al. 2018), the development of standards for smart financial contracts (Brammertz and Mendelowitz 2018), the application of the blockchain framework to insurance processes within the insurance industry (Raikwar et al. 2018), and secure authentification protocols for mobile payments (Fan et al. 2018). However, Fintech payment services still face challenges in terms of authorization, integrity, privacy, and availability, which affects consumer trust. Information technology is the premise for guaranteeing the security of data and service quality, thereby improving consumer trust even more so (Wiradinata 2018; Sarkar et al. 2020). Some studies have explored how the use of mobile digital payments influences purchasing in terms of such factors as service quality and security (Tang et al. 2021). Khoa (2021) demonstrated that both the benefits and the risks that the consumer perceives are important considerations for the users of mobile payment platforms. See Sun et al. (2021) for more detailed information on Fintech-related tendencies and topics.

With regard to accounting, digitalization leads professionals to move beyond their occupational limits. In this way, examples arise of situations in which the boundaries between professions become hazy. One revealing example was provided by Arnaboldi et al. (2017), who showed how media marketing specialists entered the area of accounting taking the lead in the management of social networks. Their study indicated that when the professionals crossed the organizational limits, the hybridization of professional roles became self-evident. In Arnaboldi et al. (2017), hybridization referred to a situation in which the actors of the organization migrated to other organizational areas, in other words, when marketing specialists entered the terrain of traditional accounting, or when accounting professionals took charge of digitalization initiatives. Social networks have relaxing effects on professional boundaries (Arnaboldi et al. 2017), the driving force being the development of digital tools and techniques. Finally, cryptocurrencies have won broad acceptance in the market and made rapid development despite their recent launches. The academic community has also made considerable efforts to investigate cryptocurrency commerce (see Fang et al. 2022).

## Research agenda

Research into the digitalization of the firm has to be continuous, because technology is a dynamic organism (Prasad and Green 2015) and, consequently, its impact on the firm is also dynamic. In this section, the emergent tendencies of digitalization are analyzed in the different areas of the firm and identified in various firms, while responding to research question RQ3.

### Theoretical contributions

The systemic review presented in this paper on digitalization in the different functional areas of the firm has highlighted that some theories have hardly been taken into account in any of the disciplines considered in this investigation that support aspects of digitalization, DT, and the digital technologies that have been analyzed. The extended technology acceptance model has been employed for the digitalization of the firm in the area of marketing, in order to estimate the degree of consumer acceptance of digitalization (Labus, 2022), although adaptive theoretical frameworks centered on the Fishbein-Ajzen behavioral-intentions model have also been used (Fishbein and Ajzen 1975; Ajzen and Fishbein 1980). Mental process, the motivation of the consumer, attitudinal and behavioral models, and the relations between the consumer and the brand are found among a number of investigations that have significantly increased (Park and Yoo 2020). However, understanding the effects of mobile technologies on the life of the digital consumer has been turned into a need for effective marketing (Vahdat et al. 2021). In the case of immersive technologies (augmented reality (AR) and virtual reality (VR)), models are needed that include consumer characteristics such as motivations, experience, and familiarity, since these attributes are what change consumer perceptions of both the risks and the benefits of real value. The theoretical progress in management has led to the generation of new theoretical models or the adaptation of existing ones when the limits of DT exceed the established theoretical models (Hanelt et al. 2021). Technological impact, compartmentalized adaptation, systemic change, and holistic co-evaluation (Hanelt et al. 2021) are perspectives that imply an organizational change associated with the generalized diffusion of digital technologies.

Although the evolution of research on digitalization on marketing as a discipline has been centered on the concept of value and its meaning for the consumer in the digital world, as well as on its components -utilitarian and hedonic values- and on joint value creation -collaborative consumption-, some interesting topics have been highlighted in this paper on the frontier between the firm and the consumer, such as value creation and co-creation, technological advances such as AI, AR and VR in apps, impacting on consumer preferences and behavior. The dominant theories, contexts, characteristics, and methodologies identified in the analysis of consumer brand personality perceptions were employed to study the perceptions of consumers towards the personality of digital brands within different digital contexts, such as websites and online stores, social media (mobiles apps and online community), new technologies (AR, mixed reality and VR), and other contexts (online games, virtual worlds and metaverse), without forgetting the interaction between the consumer and AI, such as IoT-based devices, smart objects, chatbots, intelligent assistants, service/social robots, wearables, and AI-driven algorithms/platforms. The digital channels and the use of technology in the omnichannel marketing, or the integration of channels (Hossain et al. 2020), and touchpoints (Wagner et al. 2020) are some of the aspects that have been analyzed in this review. In the retail field, we have highlighted the importance of data within the single-channel context and the proliferation of online technologies and devices that access such data on Internet. Nevertheless, entrepreneurial practices initiated in response to the pandemics have opened new horizons of research (Yaghtin et al. 2021): (1) electronic commerce platforms and satisfying the information needs of clients; (2) products and services within digital showrooms; (3) digital contents to discover new market opportunities; and (4) the data and their high quality analysis, as well as the implementation of other data-based techniques such as automatic learning methods for high-quality data presentations, are posited as acceptable practice to attract clients to the digital platform of firms.

With regard to management, while the interest of research in DT is growing, there are still considerable uncertainties as to what DT is and what it covers. The result is that previous knowledge of organizational change can be problematic as the basis for a better understanding of the phenomenon and to offer informed counsel for practice. DT is defined here as organizational change that is triggered and molded by generalized diffusion of digital technology. The content of this change encompasses a movement towards malleable organizational designs that are integrated and driven by digital business ecosystems. This content of change can be seen from four different perspectives, which include the perspectives of technological impact, compartmentalized adaptation, systemic change, and wholistic co-evolution. The perspectives vary in their contextual scope and are centered on the processes of change within the organization, but share the common characteristic of associating organizational change with the nature of digital technologies, in particular their omnipresence and the dynamics that they induce. When linking our findings with established knowledge on organizational change, the diagnosis is that DT can be better understood as continuous change that can be triggered and molded by episodic upheavals, whereas organizational change induces additional continuous changes.

Finally, the emergent trends within the disciplines of finance and accounting continue to be on the topic of Fintech. First, information technology is still essential to uphold the future development of Fintech, because the additional improvement of algorithms will promote the development of information technology. Second, AI is a critical point of research and, therefore, future research will explicitly emphasize the impact of intelligent products on AI technology in the finance industry. It is acknowledged in the methodology that a complete range of potential clients use robot-consultants and that personal and sociodemographic variables could modulate primary relations (Belanche et al. 2019). Third, digital currency, government, finances, science and technology could be the focus of future investigations (Joao 2018; Rodriguez Bolivar and Scholl 2019). In fourth place, information technology has its unique development process in different financial fields, which will have a disruptive impact on the financial markets (Hua et al. 2019). Finally, the dynamic mechanism and the social impact of Fintech in the future will continue to be the principal direction of research.

### Managerial implications

In this study, some managerial implications have been identified in the field of marketing such as the improvement of the customer experience and the ease of customer relations through immersive technologies such as VR and AR, and interactive technologies such as (AI). In the case of customer experience, although firms can rely on cognitive technologies to improve client experience in the systems of electronic commerce (Krsteva 2016), more research is needed to understand how consumer experiences can be improved when they use technological applications both in shops (Jäger and Weber 2020, van Esch et al. 2021) and in cash machines (Cui et al. 2021), and why immersive technologies within retail commerce are still insufficient (Parekh et al. 2020). Firms should also take into account the experiences of virtual consumers in multiple environments, as well as retail sales and social networks. Continuing with the analysis of the consumer, defining patterns of transcultural consumption (Alsaleh et al. 2019) is an interesting topic for firms because digitalization is global and digital firms can sell and deliver goods beyond geographical frontiers.

Firms must continue to make progress in facilitating relationships with their customers through AI as social media are turned into the most influential channel of digital marketing. Firms could therefore use a model trained for (1) social-media data analysis and visualization in real time for their interpretation; and (2) for studying and visualizing the paths that may lead to a more committed audience (Capatina et al. 2020); and, consequently, to help digital agencies to achieve greater leverage for marketing on social media.

Social networks continue their significant growth, and firms must continue to pay attention to eWOM (Bu et al. 2021; Park et al. 2021), consumer engagement (de Oliveira Santini et al. 2020; García-de-Frutos and Estrella Ramón 2021), and branding (Dabbous and Barakat 2020; McClure and Seock 2020; Schivinski et al. 2021), without forgetting source credibility. Firms have to analyze the impact of social media influencers and the influence of celebrities on their branding activities (Sokolova and Kefi 2020).

In the field of management, the managerial implications are focused on processes of change driven by the growing diffusion of AI, robotics and the Internet, and their effect on organizational design (Korte et al. 2021). The implication of Industry 4.0 technologies for business innovation is turning out to be an interesting topic for firms. Specifically, robotics and additive manufacturing can optimize the innovation of ecological processes (Liu and De Diovanni 2019); the analysis of big data can drive supply chain innovations (Hopkins 2021); and the technologies of Industry 4.0 can catalyze the innovation of the business model for servitization (Frank et al. 2019) and the circular economy (Awan et al. 2021).

This organizational change arising in particular from DT requires the adoption of three important leadership skills within the firm: awareness of DT, acceleration of DT, and harmonization of DT. Each skill involves specific leadership attributes and capabilities and firms that are certain that they have the three leadership skills within their organization are better prepared to master the challenges of DT.

First, managers need not only to observe and to react to changes, but they also need to be aware of the varied quantity of data, and the emergent digital technologies, as well as their properties. Managers must understand that these elements are rapidly evolving and must take into account that they are interlinked with aspects related to the respective characteristics of the market, the consumer, and the country. Managers must use data analysis in a proactive manner as a management tool, in order to take this complexity into account in their evaluations of the conditions of the business environment.

Second, managers require management skills to accelerate DT in the execution of the strategy relating to innovation mechanisms within a multi-dimensional framework. These skills refer to the intellectual capability to conceive processes and novel digital products as a function of internal and external resources, as well as the willingness to invest managerial attention and financial resources during times of episodic upheavals. Above all, it is important to understand that rapid execution and experimentation to learn in the market are superior to *ex ante* planning and analysis, due to the dynamic nature of the ecosystems of digital businesses, the transience of competitive advantages and positioning.

Third, managers require skills of harmonization with DT with respect to the processes of organizational change. These processes are once again related with the mechanisms within the multi-dimensional framework, but with an approach on integration. The skills of harmonization with DT include managerial skills, so that new products and digital processes can be integrated within the existing organization. It covers the union and communication between ‘physical’ and ‘digital’ units, comprising areas of synergies and areas of friction, reconciling differences, and lending attention to cultural aspects through balance and combination. The managers need to focus their attention here, in other words, to decide what to maintain and what to change in the course of DT, which depends on general opportunities and risks, as well as on each specific industry, and the nature of the changes that may be taking place.

Finally, the managerial implications in the field of finance, are focused on the Fintech sector. The rapid development of technology has had a disruptive impact on traditional financing, not only creating new opportunities for business (crowdfunding, crowdlending, digital money), but also generating a large number of uncontrollable financial risks (Bromberg et al. 2017; Duca 2000): abuse of personal data, which has been turned into one of the main concerns of both consumers and regulators (Jagtiani and John 2018); information asymmetries, and even possible systemic risks have appeared in the process of technology-driven financial innovation (Yang and Li 2018). Bermann et al. (2021) showed that external technological funding (for example, indexed funds and exchange-traded funds) improved the competitiveness of small firms and stimulated the business spirit, by reducing the complexity and the costs of financial services. In the risk financing market, financial innovation firms must reach a size and a set quantity to influence the Fintech entrepreneurial spirit in a positive manner (Kolokas et al. 2020).

The new business models of financial services are focused on client needs more than anything else, offering the possibility of instantaneous digital transactions and obtaining sustainable competitive advantage. But the advent of innovative technologies has also created operational challenges for emergent firms, designing operational strategies within the financial service from the viewpoint of the client (Choi 2021). For example, a new hybrid multiple-standards decision-making approach has been proposed to evaluate the innovation of services, with new commercial partners as the maximum priority (Zhao et al. 2019).

## Directions for future research

This systematic review of the literature on digitalization in three mature areas of the firm: marketing, management, and finance and accounting provides future lines of research.

In the field of marketing, the first topic that is shown is related with immersive technologies such as (AR), (VR), and interactive technologies such as AI. The capability of immersive technologies to improve upon the efficiency and the effectiveness of the client experience requires more detailed examination. To do so, the results of immersive technologies need to be investigated in the context of value creation for the client. The impact of technology to facilitate client relations is another frontier of investigation that can be explored. Specifically, IA is a technology quite new in comparison with others such as AR and VR, which means that analyzing the impact of IA on digital consumer behavior patterns is a very broad area of study. The fusion of AI and marketing will certainly grow (Pitt et al. 2018; Wirth 2018; Vishnoi and Bagga 2019) in such a way that investigating the consumer journey facilitated by AI technology can help academia to obtain information on the benefits of IA technology. Some other topics for research that researchers have identified in this field are as follows: to use smart algorithms for in-depth study of automated marketing concepts (Dumitriu and Popescu 2020) and to observe how multichannel AI systems can help to create a lean or a more intelligent technology (Cosmin Tănase 2018).

Future research must seek a more multicultural approach to define the specific differences of each country in terms of the use of online channels, the adoption of technology, and the commitment of digital channels to completely understand digital consumption.

Environmental problems, greater awareness of them among people and the recent COVID-19 health crisis has awakened interest in such topics as sustainability, wellbeing, and value creation. They especially highlighted the studies within the frame of sustainable consumption that concerned their work, principally with collaborative consumption in a digital context, and online rental activities (Lee and Chow 2020; Lee and Huang 2020; Luo et al. 2020).

The possible future of social media according to marketing-related questions and its impact on the individual, the firm and public policies is another area for future research that will be addressed in coming years. Finally, another subject that is closely associated with social media studies is source credibility. Specifically, the investigation will be centered on analyzing the impact of social media influencers and the impact of celebrities on firm branding activities (Sokolova and Kefi 2020).

In the field of management and managerial skills, continued research on DT is necessary through the perspective of systemic changes, holistic co-evaluation, and compartmentalized adaptation to advance towards the development of awareness, acceleration, and harmonization of DT, respectively. Also, more research on all those aspects of intra-organizational integration and innovation as drivers of malleable organizational designs is still needed. For example, when describing strategies and steps on how organizations can guide their efforts to achieve DT, it is also proposed that future investigations on DT be approached through a complementary ethical perspective focused on three aspects: the balance between organizational effort, ethics, and value co-creation. Finally, a whole research gap is opened on the implications of Industry 4.0 technologies in business innovation (Oztemet and Gursev 2020).

In finance, the topics that have been identified for research in this study contribute to various currents of thought in the literature, including the emergent literature on Fintech, and the literature on entrepreneurship, innovation, and international business. In the first place, the field of Fintech entrepreneurship is an emergent area that is fundamental to promote entrepreneurship and the production of innovative technologies (Álvarez et al. 2016). Specifically, external factors can influence the distribution of the quality of entrepreneurship when the entry of low-quality firms is reduced (Khanin et al. 2022; Ritala et al. 2021). Studies have compared the differences between reward crowdfunding and capital crowdfunding in the development of Fintech, which can help to comprehend the workings of the entrepreneurial spirit better (Troise et al. 2021). In addition, Fintech as a strategy in the digital age, is presented as an interesting research topic, since it is having an impact on cross-border commerce, causing changes in the nature of organizations and in-service businesses (for example, commercial contracts and currency trading). Finally, the business model of digital money is another of the focuses of future research (Joao 2018; Rodriguez Bolivar and Scholl 2019), since mobile payments have almost pervaded the daily life of many people, with wide coverage, and innovative payment methods that show constant vitality (Sun et al. 2022).

In summary, the challenges that the digitalization of firms, institutions and society in general presents in this day and age are cybersecurity and user privacy (Wylde et al. 2022). Specifically, maintaining a balance between the anonymity of users and the traceability of the transaction (Fujitani et al. 2021); guaranteeing security and privacy of financial data in the face of a tendency towards open banking (Liao et al. 2022), financial scamming, and hacking (Zhao 2021). In the case of SMEs, what remains for them to do is to employ the technology as a means of transformation to confront a competitive, complex, and uncertain business environment (Rozmi et al. 2021) All these challenges are presented as incipient lines of investigation, together with others such as the processing of information obtained with different technologies from firms and their rapid response to market fluctuations (Li et al. 2021). The way in which automatic learning algorithms function and the extent to which they can affect organizational decisions and actions, being at the root of tactical and strategic errors, also remain to be studied in depth.

## Conclusions and limitations

Over the past decade and above all, as from 2020, an increase in the number of publications on digitalization, digital technologies, and DT have been seen. Although this paper is centered on the more mature areas of the firm, the literature is limited. In this paper, a detailed analysis of current achievements in investigation of digitalization has been performed, taking into account the most relevant publications. A systematic review has been conducted of the literature based on the sample of 119 high-quality peer-reviewed review articles in the fields of management, marketing, and finance and accounting.

In the discipline of marketing, five principal research tendencies have been identified in current investigations: (1) the performance of digital marketing from the perspective of the consumer centered on its adoption and engagement; (2) the management of social networks as a marketing strategy; (3) knowledge of consumer behavior through big-date technology; (4) the impact of mass-media campaigns through digital technologies; and (5) the customer journey via online outlets.

In the case of management, three principal tendencies have been highlighted in this study: (1) information management and performance, so that it is converted into knowledge for organizations; (2) the design of flexible organizations to adapt themselves to their business environment; (3) the impact of Internet technologies and digitalization on logistics and supply channel management.

Finally, in the discipline of finances and accounting, a tendency may be highlighted for each discipline. In finances, the principal research tendency is to continue inquiring into technologies such as blockchain applied to financial services, whereas in accounting the impact of cryptocurrency may be highlighted in accounting systems and risk management.

As a final conclusion, this paper may be considered to have: (1) provided the most complete and updated review of digitalization from a global perspective, summarizing the current state of knowledge within an integrated framework; (2) reduced the complexity of digitalization by offering structure and clarity; and (3) advanced links between digitalization and established points of view in the literature on management, marketing, and finance and accounting.

Our study has some limitations that can be identified in the steps of our research process. First, given that our study used a particular database, some articles may have been overlooked in the data-compilation process. Second, the filters applied in the data-analysis process might have omitted some relevant studies, due to our choices of cut-off decisions (for example, with respect to the time frames). In addition, the process of encoding the articles was manual and might therefore have been affected by subjectivity, although we sought to avoid that bias by performing multiple encoding routines and various rounds of checking the encoding. Third, with respect to the summarization, it is accepted that there might be valuable alternatives to an ‘entry-process-exit’ model (Edmondson and Mcmanus 2007) as a meta-structure. In the light of these limitations, we encourage other researchers to enlarge and to improve upon our findings, employing different sources and analytical approaches.

## Appendix

Appendix 1: Corpus of review articles analyzed in the study.

## References

[CR1] Ajzen I, Fishbein M (1980). Understanding attitudes and predicting social behavior.

[CR2] Alos-Simo L, Verdu-Jover A, Gomez-Gras J (2017). How transformational leadership facilitates e-business adoption. Ind Manag Data Syst.

[CR3] Alsaleh DA, Elliott MT, Fu FQ, Thakur R (2019). Cross-cultural differences in the adoption of social media. J Res Interact Market.

[CR4] Alvarez SA, Audretsch D, Link AN (2016). Advancing our understanding of theory in entrepreneurship. Strat Entrepren J.

[CR5] Alzamora-Ruiz J, Fuentes-Fuentes MD, Martinez-Fiestas M (2021). Together or separately? Direct and synergistic effects of Effectuation and Causation on innovation in technology-based SMEs. Int Entrep Manag J.

[CR6] Ardito L, Petruzzelli AM, Panniello U, Garavelli AC (2019). Towards industry 4.0: mapping digital technologies for supply chain management-marketing integration. Bus Process Manag J.

[CR7] Arnaboldi M, Busco C, Cuganesan S (2017). Accounting, accountability, social media and big data: revolution or hype?. Acc Audit Acc J.

[CR8] Aström J, Reim W, Parida V (2022). Value creation and value capture for AI business model innovation: a three–phase process framework. Rev Manag Sci.

[CR9] Atkinson L (2013). Smart shoppers? Using QR codes and ‘green’ smartphone apps to mobilize sustainable consumption in the retail environment. Int J Consum Stud.

[CR10] Awan U, Sroufe R, Shahbaz M (2021). Industry 4.0 and the circular economy: a literature review and recommendations for future research. Bus Strat Environ.

[CR11] Bardhi F, Eckhardt GM (2017). Liquid consumption. J Consum Res.

[CR12] Barney J (1991). Firm resources and sustained competitive. J Manag.

[CR13] Bhatia MS, Kumar S (2022). Critical success factors of industry in automotive manufacturing industry. IEEE Trans Eng Manage.

[CR14] Belanche D, Casaló LV, Flavián C (2019). Artificial intelligence in fintech: understanding robo-advisors adoption among customers. Ind Manag Data Syst.

[CR15] Benlian A, Kettinger WJ, Sunyaev A, Winkler TJ (2018). Special section: the transformative value of cloud computing: a decoupling, platformization, and recombination theoretical framework. J Manag Inform Syst.

[CR16] Berger J, Milkman KL (2012). What makes online content viral?. J Market Res.

[CR17] Berman A, Cano-Kollmann M, Mudambi R (2021). Innovation and entrepreneurial ecosystems: Fintech in the financial services industry. Rev Manag Sci.

[CR18] Biggi G, Giuliani E (2020). The noxious consequences of innovation what do we know?. Ind Innovat.

[CR19] Bilro RG, Loureiro SMC, Guerreiro J (2019). Exploring online customer engagement with hospitality products and its relationship with involvement, emotional states, experience and brand advocacy. J Hospit Market Manag.

[CR20] Bitran G, Gurumurthi S, Sam S (2007). The need for third-party coordination in supply chain governance. MIT Sloan Manag Rev.

[CR21] Bleier A, Harmeling CM, Palmatier RW (2019). Creating effective online customer experiences. J Market.

[CR22] Blom A, Lange F, Hess RL (2017). Omnichannel-based promotions’ effects on purchase behavior and brand image. J Retailing Consum Serv.

[CR23] Boitan IA, Stefoni SE (2022). Digitalization and the shadow economy; impact assessment and policy implications for EU countries. East. Eur Econ Early Access: Jul 2022.

[CR24] Bouncken RB, Kraus S (2022). Entrepreneurial ecosystems in an interconnected world: emergence, governance and digitalization. Rev Manag Sci.

[CR25] Bouncken RB, Kraus S, Roig-Tierno N (2021). Knowledge- and innovation-based business models for future growth: digitalized business models and portfolio considerations. Rev Manag Sci.

[CR26] Brammertz W, Mendelowitz AI (2018). From digital currencies to digital finance: the case for a smart financial contract standard. J Risk Finance.

[CR27] Bromberg L, Godwin A, Ramsay I (2017). Cross-border cooperation in financial regulation: crossing the fintech bridge. Cap Mark Law J.

[CR28] Bronner F, Kuijlen T (2007). The live or digital interviewer-a comparison between CASI, CAPI and CATI with respect to differences in response behavior. Int J Market Res.

[CR29] Brynjolfsson E, Hu Y, Rahman M (2013). Competing in the age of omnichannel retailing. MIT Sloan Manag Rev.

[CR30] Bu Y, Parkinson J, Thaichon P (2021). Digital content marketing as a catalyst for e-WOM in food tourism. Australas Market J.

[CR31] Cai L (2018). Disruption of financial intermediation by fintech: a review on crowdfunding and blockchain. Acc Finance.

[CR32] Capatina A, Kachour M, Lichy J, Micu A, Micu A, Codignola F (2020). Matching the future capabilities of an artificial intelligence-based software for social media marketing with potential users’ expectations. Technol Forecast Soc Change.

[CR33] Chan YE, Krishnamurthy R, Sadreddin A (2022). Digitally-enabled university incubation processes. Technovation.

[CR34] Chartterjee S, Chaudhuri R, Vrontis D, Basile G (2022). Digital transformation and entrepreneurship process in SMEs of India: a moderating role of adoption of AI-CRM capability and strategic planning. J Strat Manag.

[CR35] Chaudhary S, Dhir A, Ferraris A, Bertoldi B (2021). Trust and reputation in family businesses: a systematic literature review of past achievements and future promises. J Bus Res.

[CR36] Childers TL, Carr CL, Peck J, Carson S (2001). Hedonic and utilitarian motivations for online retail shopping behavior. J Retailing.

[CR37] Choi M (2021). The effect of perceived customer orientation on the customer intention in fintech service: focused on the technology acceptance model. Inf Syst Rev.

[CR38] Chu SC, Kim J (2018). The current state of knowledge on electronic word-of-mouth in advertising research. Int J Advert.

[CR39] Cortet M, Rijks T, Nijland S (2016). PSD2: the digital transformation accelerator for banks. J Payments Strategy & Systems.

[CR40] Cosmin TĂNASE (2018). Artificial intelligence: optimizing the experience of digital marketing Cosmin TĂNASE. Romanian Distribution Committee Magazine.

[CR41] Cui YG, van Esch P, Jain SP (2021). Just walk out: the effect of AI-enabled checkouts. Eur J Market.

[CR42] Dabbous A, Barakat KA (2020). Bridging the online offline gap: assessing the impact of brands’ social network content quality on brand awareness and purchase intention. J Retailing Consum Serv.

[CR43] Dalenogare LS, Benitez GB, Ayala NF, Frank AG (2018). The expected contribution of industry 4.0 technologies for industrial performance. Int J Prod Econ.

[CR44] De Oliveira Santini F, Ladeira WJ, Pinto DC, Herter MM, Sampaio CH, Babin BJ (2020). Customer engagement in social media: a framework and meta-analysis. J Acad Market Sci.

[CR45] Dellaert BG (2019). The consumer production journey: marketing to consumers as co-producers in the sharing economy. J Acad Market Sci.

[CR46] Dellarocas C (2003). The digitization of word of mouth: promise and challenges of online feedback mechanisms. Manag Sci.

[CR47] Dery K, Sebastian IM, van der Meulen N (2017). The digital workplace is key to digital innovation. MIS Q Exec.

[CR48] Dewan R, Jing B, Seidmann A (2003). Product customization and price competition on the internet. Manag Sci.

[CR49] Dinner IM, Heerde Van HJ, Neslin SA (2014). Driving online and offline sales: the cross-channel effects of traditional, online display, and paid search advertising. J Market Res.

[CR50] Dou W, Linn R, Yang S (2001). How smart are ‘smart banners’?. J Advert Res.

[CR51] Downes L, Nunes P (2013). The big idea – the big bang disruption. Harv Bus Rev.

[CR52] Duca LM (2000). Financial technology shocks and the case of the missing m2. J Money Credit Bank.

[CR53] Dumitriu D, Popescu MAM (2020). Artificial intelligence solutions for digital marketing. Procedia Manuf.

[CR54] Edmondson A, McManus S (2007). Methodological fit in management field research. Acad Manag Rev.

[CR55] El Sawy OA, Malhotra A, Park Y, Pavlou PA (2010). Research commentary – seeking the configurations of digital ecodynamics: it takes three to tango. Inf Syst Res.

[CR56] El Sawy OA, Pereira F (2013). Business modelling in the Dynamic Digital Space.

[CR57] Emmer ET (2018). Glofs in the WoS: Bibliometrics, geographies and global trends of research on glacial lake outburst floods (web of science, 1979–2016). NHESS.

[CR58] Fan K, Li H, Jiang W, Xiao C, Yang Y (2018). Secure authentication protocol for mobile payment. Tsing-hua Sci Technol.

[CR59] Fang F, Ventre C, Basios M, Kanthan L, Martinez-Rego, Wu F, Li LB (2022). Cryptocurrency trading: a comprehensive survey. Financial Innov.

[CR60] Fishbein M, Ajzen I (1975). Belief, attitude, intention, and behavior: an introduction to theory and research.

[CR61] Flavián C, Gurrea R, Orús C (2016). Choice confidence in the webrooming purchase process: the impact of online positive reviews and the motivation to touch. J Consum Behav.

[CR62] Floyd K, Freling R, Alhoqail S, Cho HY, Freling T (2014). How online product reviews affect retail sales: a meta-analysis. J Retailing.

[CR63] Frank AG, Mendes GH, Ayala NF, Ghezzi A (2019). Servitization and industry 4.0 convergence in the digital transformation of product firms: a business model innovation perspective. Technol Forecast Soc Change.

[CR64] Fujitani T, Emura K, Omote K (2021) A privacy-preserving enforced bill collection system using smart contracts. In: 16th Asia Joint Conference on Information Security (ASIAJCIS 2021), pp 51–60

[CR65] Gallarza MG, Gil-Saura I, Holbrook MB (2011). The value of value: further excursions on the meaning and role of customer value. J Consum Behav.

[CR66] García-de-Frutos N, Estrella-Ramón A (2021). You absolutely (don’t) need this! Examining differences on customer engagement components for (anti) haul youtubers’ videos. J Rese Interact Market.

[CR67] Ghobakhloo M (2020). Industry 4.0, digitalization, and opportunities for sustainability. J Clean Prod.

[CR68] Godes D, Mayzlin D (2004). Using online conversations to study word-of-mouth communication. Market Sci.

[CR69] Gossling S, Scott D, Hall CM (2021). Pandemics, tourism and global change: a rapid assessment of COVID-19. J Sustain Tourism.

[CR70] Gray P, El Sawy OA, Asper G, Thordarson M (2013). Realizing strategic value through center-edge digital transformation in consumer-centric industries. MIS Q Exec.

[CR71] Guo YM, Huang ZL, Guo J, Li H, Guo XR, Nkeli MJ (2019). Bibliometric analysis on Smart Cities Research. Sustainability.

[CR72] Hanelt A, Bohnsack R, Marz D, Marante CA (2021). A systematic review of the literature on digital transformation: insights and implications for strategy and organizational. Change. J Manag Stud.

[CR73] Hess T, Matt C, Belina A, Wiesböck F (2016). Options for formulating a digital transformation strategy. MIS Q Exec.

[CR74] Hoddap D, Hanelt A (2022). Interoperability in the era of digital innovation: an information systems research agenda. J Informa Technol.

[CR75] Hollebeek LD, Juric B, Tang W (2017). Virtual brand community engagement practices: a refined typology and model. J Servi Market.

[CR76] Hopkins JL (2021). An investigation into emerging industry 4.0 technologies as drivers of supply chain innovation in Australia. Comput Ind.

[CR77] Hossain TMT, Akter S, Kattiyapornpong V, Dwivedi Y (2020). Reconceptualization integration quality dynamics for omnichannel marketing. Ind Market Manag.

[CR78] Hua X, Huang Y, Zheng Y (2019). Current practices, new insights, and emerging trends of financial technologies. Ind Manag Data Syst.

[CR79] Hwang J, Griffiths MA (2017). Share more, drive less: Millennials value perception and behavioral intent in using collaborative consumption services. J Consum Market.

[CR80] Jacobides M, Cennamo C, Gawer A (2018). Towards a theory of ecosystems. Strat Manag J.

[CR81] Jäger AK, Weber A (2020). Increasing sustainable consumption: message framing and in-store technology. Int J Retail Distrib Manag.

[CR82] Jagtiani J, John K (2018). Fintech: the impact on consumers and regulatory responses. J Econ Bus.

[CR83] Joao JA (2018). Blockchain and the potential of new business models: a systematic mapping. Rev Gest Proj.

[CR84] Khanin D, Rosenfield R, Mahto R, Singhal C (2022). Barriers to entrepreneurship: opportunity recognition vs. opportunity pursuit. Rev Manag Sci.

[CR85] Khoa DB (2021). The impact of the personal data disclosure’s tradeoff on the trust and attitude loyalty in mobile banking services. J Promot Manag.

[CR86] King RA, Racherla P, Bush VD (2014). What we know and don’t know about online word-of-mouth: a review and synthesis of the literature. J Interact Mark.

[CR87] Kolokas D, Vanacker T, Veredas D, Zahra SA (2020). Venture capital, credit, and fintech start-up formation: a cross-country study. Entrepren Theory Pract.

[CR88] Kopalle P, Kumar V, Subramaniam M (2020). How legacy firms can embrace the digital ecosystem via digital customer orientation. J Acad Market Sci.

[CR89] Korte A, Tiberius V, Brem A (2021). Internet of things (IoT) technology research in business and management literature: results from a co-citation analysis. J Theor Appl Electronic Commerce Res.

[CR90] Kraus S, Breier M, Lim WM, Ferraris A, Fernandes C, Ferreira JJ (2022). Literature reviews and independent studies: guidelines for academic practice. Rev Manag Sci.

[CR91] Kraus S, Jones P, Kailer N, Weinmann A, Chaparro-Benegas N, Roig-Tierno N (2021) Digital transformation: an overview of the current state of the art of research. 10.1177/21582440211047576. SAGE Open July-September

[CR92] Kraus S, Breier M, Dasí-Rodríguez S (2020). The art of crafting: a systematic literature review in entrepreneurship research. Int Entrep Manag J.

[CR93] Krsteva MST (2016). Artificial intelligence in marketing and advertising. Int J Science and Arts.

[CR94] Kurniawan TA, Othman MHD, Hwang GH, Gikas P (2022). Unlocking digital technologies for waste recycling in industry 4.0 era: a transformation towards a digitalization-based circular economy in Indonesia. J Clean Prod 357 Article Number: 131911.

[CR95] Labus P, Jelovac D (2022). Customer acceptance of digitalization of hotel restaurants: applying an extended technology acceptance model. Acta Turística.

[CR96] Lamberton C, Stephen AT (2016). A thematic exploration of digital, social media, and mobile marketing: Research evolution from 2000 to 2015 and an agenda for future inquiry. J Market.

[CR97] Lecinski J (2011). Winning the zero moment of truth: ZMOT. Zero Moment of Truth.

[CR98] Lee SH, Chow PS (2020). Investigating consumer attitudes and intentions toward online fashion renting retailing. J Retailing Consum Servi.

[CR99] Lee SH, Huang R (2020). Consumer responses to online fashion renting: exploring the role of cultural differences. Int J Retail Distrib Manag.

[CR100] Legner C, Eymann T, Hess T, Matt C, Böhmann T, Drews P, Mädche A, Urbach N, Ahlemann F (2017). Digitalization: opportunity and challenge for the business and information systems engineering community. Bus Inf Syst Eng.

[CR101] Lemon KN, Verhoef PC (2016). Understanding customer experience throughout the customer journey. J Market.

[CR102] Li D, Fast-Berglund Ã, Paulin D (2019). Current and future industry 4.0 capabilities for information and knowledge sharing. Int J Advanced Manufacturing Technol.

[CR103] Li H, Wu Y, Cao D, Wang Y (2021). Organisational mindfulness towards digital transformation as a prerequisite of information processing capability to achieve market agility. J Bus Res.

[CR104] Liao CH, Guan XO, Cheng JH, Yuan SM (2022). Blockchain-based identity management and access control framework for open banking ecosystem, Future Generation Computer systems. Inter J Escience.

[CR105] Liu B, De Giovanni P (2019). Green process innovation through industry 4.0 technologies and supply chain coordination. Annals of Operations Res.

[CR106] Luo B, Sun Y, Shen J, Xia L (2020). How does green advertising skepticism on social media affect consumer intention to purchase green products?. J Consum Behav.

[CR107] Marinakis YD, White R (2022). Hyperinflation potential in commodity-currency trading systems: implications for sustainable development. Sustain Technol and Entrepren.

[CR108] Marshakova I (1973). System of document connections based on references. Nauchn Tech Inform.

[CR109] Massaro M, Dumay J, Guthrie J (2016). On the shoulders of giants: undertaking a structured literature review in accounting. Acc Audit Acc J.

[CR110] McClure C, Seock YK (2020). The role of involvement: investigating the effect of brand’s social media pages on consumer purchase intention. J Retailing Consum Servi.

[CR111] Micu A, Capatina A, Micu AE (2018). Exploring artificial intelligence techniques’ applicability in social media marketing. J Emerg Trends Mark Manage.

[CR112] Moher D, Shamserr L, Clarke M (2015). Preferred reporting items for systematic review and metanalysis protocols (PRISMA-P) 2015 statement. Syst Rev.

[CR113] Möhlmann M (2015). Collaborative consumption: determinants of satisfaction and the likelihood of using a sharing economy option again. J Consum Behav.

[CR114] Muhuri PK, Shukla AK, Abraham A (2019). Industry 4.0: a bibliometric analysis and detailed overview. Eng Appl Artif Intell.

[CR115] Nambisan S (2017). Digital entrerpreneurship: toward a digital technology perspective of entrepreneurship. Enterpren Theor Pract.

[CR116] Nasiri M, Saunila M, Ukko J (2022). Digital orientation, digital maturity, and digital intensity: determinants of financial success in digital transformation settings. Int J Oper Prod Manage.

[CR117] Norberg PA, Horne DR, Horne DA (2007). The privacy paradox: personal information disclosure intentions versus behaviors. J Consum Aff.

[CR118] Novak TP, Hoffman DL, Yung YF (2000). Measuring the customer experience in online environments: a structural modeling approach. Market Sci.

[CR119] Nyagadza B (2022). Sustainable digital transformation for ambidextrous digital firms: a systematic literature review and future research directions. Sustain Technol Entrepreneurship.

[CR120] Oztemet E, Gursev S (2020). Literature review of industry 4.0 and related technologies. J Intelli Manuf.

[CR121] Pagani M (2013). Digital business strategy and value creation: framing the dynamic cycle of control points. MIS Q.

[CR122] Parekh P, Patel S, Patel N, Shah M (2020). Systematic review and meta-analysis of augmented reality in medicine, retail, and games. Visual Comput Ind Biomed Art.

[CR123] Park J, Hyun H, Thavisay T (2021). A study of antecedents and outcomes of social media WOM towards luxury brand purchase intention. J Retailing Consum Servi.

[CR124] Park M, Yoo J (2020). Effects of perceived interactivity of augmented reality on consumer responses: a mental imagery perspective. J Retailing Consum Servi.

[CR125] Parker G, van Alstyne M, Jiang X (2017). Platform ecosystems: how developers invert the firm. MIS Q.

[CR126] Petit O, Velasco C, Spence C (2019). Digital sensory marketing: integrating new technologies into multisensory online experience. J Interact Marketing.

[CR127] Pitt C, Eriksson T, Dabirian A, Vella J (2018). May elementary, my dear watson: the use of artificial intelligence in marketing research: an abstract [Conference session]. Acad Market Sci.

[CR128] Post C, Sarala R, Gatrell C, Prescott J (2020). Advancing theory with review articles. J Manag Stud.

[CR129] Prasad A, Green P (2015). Governing cloud computing services: reconsideration of IT governance structures. Int J Account Inf Syst.

[CR130] Qiu M, Gai K, Zhao H, Liu M (2018) Privacy-preserving smart data storage or financial industry in cloud computing. In: Paper presented at the 2nd IEEE international symposium on security and privacy in social networks and big data (IEEE Social Sec), Fiji. 10.1002/cpe.4278

[CR131] Quach S, Thaichon P, Martin KD, Eaven S, Palmatier RW (2022). Digital technologies: tensions in privacy and data. J Acad Market Sci.

[CR132] Raff S, Wentzel D, Obwegeser N (2020). Smart products: conceptual review, synthesis and research directions. J Prod Innov Manag.

[CR133] Raikwar M, Mazumdar S, Ruj S, Gupta SS, Chattopadhyay A, Lam KY (2018) A blockchain framework for insurance processes. In: Paper presented at the 9th IFIP international conference on new technologies, mobility and security (NTMS), Paris, France

[CR134] Riaz z, Ray P, Ray S (2022). The impact of digitalization on corporate governance in Australia. J Bus Res.

[CR135] Ribeiro-Navarrete S, Saura JR, Palacios-Marqués D (2021). Towards a new era of mass data collection: assessing pandemic surveillance technologies to preserve user privacy. Technol Forecast Soc Change.

[CR136] Richter Ch, Kraus S, Brem A, Durst S, Giselbrecht C (2017). Digital entrepreneurship: innovative business models for the sharing economy. Creat Innov Manag.

[CR137] Ritala P, Baiyere A, Hughes M, Kraus S (2021). Digital strategy implementation: the role of individual entrepreneurial orientation and relational capital. Technol Forecast Soc Change.

[CR138] Rozmi A, Nohuddin PNE, Hadi ARA, Bakar MIA (2021). Identifying small and medium enterprise smart entrepreneurship training framework components using thematic analysis and expert review. Int J Advanced Computer Sci and Applications.

[CR139] Rubio-Andrés M, Ramos-González MM, Sastre-Castillo MA (2022). Driving innovation management to create shared value and sustainable growth. Rev Manag Sci.

[CR140] Sanchez M, Exposito E, Aguilar J (2020). Industry 4.0: survey from a system integration perspective. Int J Computer Integrated Manuf.

[CR141] Sanchez-Riofrio AM, Lupton NC, Rodriguez-Vasquez JG (2022). Does market digitalization always benefit firms? The latin american case. Manage Decis.

[CR142] Sarkar S, Chauhan S, Khare A (2020). A meta-analysis of antecedents and consequences of trust in mobile commerce. Int J Inf Manage.

[CR143] Sarma S, Khurana MK (2022). A era of digitalization: mobile banking adoption in India. J Sci Technol Policy Manage Early Access: Sept 2022.

[CR144] Schivinski B, Munting DG, Pontes HM, Lukasik P (2021). Influencing COBRAs: the effects of brand equity on the consumer’s propensity to engage with brand-related content on social media. J Strat Market.

[CR145] Secinaro S, Calandra D, Lanzalonga F, Ferraris A (2022). Electric vehicles’ consumer behaviours: mapping the field and providing a research agenda. J Bus Res.

[CR146] Shamseer L, Moher D, Clarke M, Ghersi D, Liberati A, Petticrew N, Shekelle P, Steart LA, the PRISMA-P group (2015). Preferred reporting items for systematic review and meta-analysis protocols (PRISMA-P) 2015: elaboration and explanation. BMJ (online).

[CR147] Sia SK, Soh c, Weill P (2016). How DBS bank pursued a digital business strategy. MIS Q Exec.

[CR148] Škare M, Blanco-Gonzalez-Tejero C, Crecente F, del Val MT (2022). Scientometric analysis on entrepreneurial skills-creativity, communication, leadership: how strong is the association?. Technol Forecast Soci Change.

[CR149] Snyder H (2019). Literature review as a research methodology: an overview and guidelines. J Bus Res.

[CR150] Sokolova K, Kefi H (2020). Instagram and YouTube bloggers promote it, why should I buy? How credibility and parasocial interaction influence purchase intentions. J Retailing Consum Servi.

[CR151] Somohano-Rodríguez FM, Madrid-Guijarro A, Lopez-Fernandez JM (2022). Does industry 4.0 really matter for SME innovation?. J Small Bus Manage.

[CR152] Statista (2022) Nominal GDP driven by digitally transformed and other enterprises worldwide 2018–2023. https://www.statista.com/statistics/1134766/nominal-gdp-driven-by-digitally-transformed-enterprises/

[CR153] Stocchi L, Pourazad N, Micahellidou N, Tanusondjaa A, Harrigan P (2022). Marketing research on mobile apps: past, present and future. J Acad Market Sci.

[CR154] Sun Y, Li SH, Wang R, Chau KY, Hong L, Ip YK, Yan W (2021). Fintech: From budding to explosion an overview of the current state of research. Rev Managl Sci.

[CR155] Teubner RA, Stockhinger J (2020). Literature review: understanding information systems strategy in the digital era. J Strat Inform Syst.

[CR156] Tiwari S (2020). Supply chain integration and industry 4.0: a systematic literature review. Benchmarking-An Int J.

[CR157] Thorseng AA, Grisot M (2017). Digitalization as institutional work: a case of designing a tool for changing diabetes care. Inf Technol People.

[CR158] Thompson DF, Walker CK (2015). A descriptive and historical review of bibliometrics with applications to medical sciences. Pharmacother J Hum Pharmacol Drug Ther.

[CR159] Tranfield D, Denyer D, Smart P (2003). Towards a methodology for developing evidence-informed management knowledge by means of systematic review. Br J Manag.

[CR160] Troise C, Matricano D, Candelo E, Sorrentino M (2021). Entrepreneurship and fintech development: comparing reward and equity crowdfunding. Meas Bus Excell.

[CR161] Troise C, Tani M, Matricano D, Ferrara E (2022). Guest editorial: Digital transformation, strategies management and entrepreneurial process: dynamics, challenges and opportunities. J Strat Manage.

[CR162] Tucker CE (2014). Social networks, personalized advertising, and privacy controls. J Market Rese.

[CR163] Uman LS (2011). Systematic reviews and meta-analyses. J Can Acad Child Adolesc Psychiatry.

[CR164] Vahdat A, Alizadeh A, Quach S, Hamelin N (2021). Would you like to shop via mobile app technology? The technology acceptance model, social factors and purchase intention. Australas Market J.

[CR165] Van de Ven A, Poole M (1995). Explaining development and change in organizations. Acad Manag Rev.

[CR166] Van Eck NJ, Waltman L (2010). Software survey: VOSviewer, a computer program for bibliometric mapping. Scientometr.

[CR167] Van Eck N, Waltman L (2020). VOSviewer Manual for VOSviewer Version 1.6. 14.

[CR168] Van Esch P, Cui Y, Jain SP (2021). Self-efficacy and callousness in consumer judgments of AI-enabled checkouts. Psychol Market.

[CR169] Van Laer T, Edson Escalas J, Ludwig S, Van Den Hende EA (2019). What happens in Vegas stays on TripAdvisor? A theory and technique to understand narrativity in consumer reviews. J Consum Rese.

[CR170] Venkatesh V, Thong JYL, Xu X (2012). Consumer acceptance and use of information technology: extending the unified theory of acceptance and use of technology. MIS Q.

[CR171] Venkatesan R, Mehta K, Bapna R (2007). Do market characteristics impact the relationship between retailer characteristics and online prices?. J Retailing.

[CR172] Verhoef PC, Kannan PK, Inman JJ (2015). From multichannel retailing to omni-channel retailing: introduction to the special issue on multichannel retailing. J Retailing.

[CR173] Verma P, Kumar V, Daim T, Sharma NK, Mittal A (2022). Identifying and prioritizing impediments of industry 4.0 to sustainable digital manufacturing: a mixed method approach. J Clean Prod.

[CR174] Vial G (2019). Understanding digital transformation: a review and a research agenda. J Strat Inform Syst.

[CR175] Vishnoi SK, Bagga T (2019). Artificial intelligence enabled marketing solutions: a review. Indian J Econ Bus.

[CR176] Vrontis D, Christofi M, Katsikeas CS (2020). An assessment of the literature on cause-related marketing: implications for international competitiveness and marketing research. Inter Market Rev.

[CR177] Wagner G, Schramm-Klein H, Steinmann S (2020). Online retailing across e-channels and e-channel touchpoints: empirical studies of consumer behavior in the multichannel e-commerce environment. J Bus Res.

[CR178] Weichert M (2017). The future of payments: how FinTech players are accelerating customer-driven innovation in financial services. J Paym Strat Syst.

[CR179] Wirtz BW, Weyerer JC, Heckeroth JK (2022). Digital disruption and digital transformation: A strategic integrative framework. Int J Innov Manage.

[CR180] Wen HW, Om Zhong, Lee CC (2022). Digitalization, competition strategy and corporate innovation: evidence from Chines manufacturing listed companies. Int Rev Financial Anal.

[CR181] Wylde V, Rawindaran N, Platts J (2022). Cybersecurity, data privacy and blockchain: a review. SN Comput Sci.

[CR182] Witschel D, Baumann D, Voigt KI (2022). How manufacturing firms navigate through stormy waters of digitalization: the role of dynamic capabilities, organizational factors and environmental turbulence for business model innovation. J Manage Organization.

[CR183] Windasari NA, Kusumawati N, Larasati N, Amelia RP (2022). Digital-only banking experience: insights from gen Y and gen Z. J Innov Knowl.

[CR184] Wiradinata T (2018) Mobile payment services adoption: The role of perceived technology risk. In: Paper presented at the international conference on orange technologies. (ICOT), Bali, Indonesia

[CR185] Wirth N (2018). Hello marketing, what can artificial intelligence help you with?. Int J Market Res.

[CR186] Yaghtin S, Safarzadeh H, Z and M.K, (2021). B2B digital content marketing in uncertain situations: a systematic review. J Bus Ind Market.

[CR187] Yang D, Li M (2018). Evolutionary approaches and the construction of technology-driven regulations. Emerg Mark Financ Trade.

[CR188] Yang Z, Peterson RT (2004). Customer perceived value, satisfaction, and loyalty. The role of switching costs. Psychol Market.

[CR189] Yeow A, Soh C, Hansen R (2017). Aligning with new digital strategy: a dynamic capabilities approach. J Strat Inform Syst.

[CR190] Yi Y, Gong T (2008). The electronic service quality model: the moderating effect of customer self-efficacy. Psychol Market.

[CR191] Yoo WS, Lee E (2011). Internet channel entry: a strategic analysis of mixed channel structures. Market Sci.

[CR192] Yoo YJ, Henfridsson O, Lyytinen K (2010). The new organizing logic of digital innovation: an agenda for information systems research. Inf Syst Res.

[CR193] Yoo Y, Lyytinen K, Boland R, Berente H, Gaskin J, Schutz D, Srinivasan N (2010). The next wave of digital innovation: Opportunities and challenges. Rep Res Workshop.

[CR194] Yoo Y, Boland RJ, Lyytinen K, Majchrzak A (2012). Organizing for innovation in the digitized world. Organ Sci.

[CR195] Yu D, Liao H (2016). Visualization and quantitative research on intuitionistic fuzzy studies. J Intell Fuzzy Syst.

[CR196] Zhao J (2021). Efficiency of corporate debt financing based on machine learning and convolutional neural network. Microprocessors Microsyst.

[CR197] Zhao Q, Tsai PH, Wang JL (2019). Improving financial service innovation strategies for enhancing China’s banking industry competitive advantage during the fintech revolution: a hybrid MCDM model. Sustainability.

[CR198] Zeng HX, Ran HX, Zhou Q, Jin YL, Cheng X (2022). The financial effect of firm digitalization: evidence from China. Technol Forecast Soci Change.

[CR199] Zhou W, Chen J, Huang Y (2019). Co-citation analysis and burst detection on financial Bubbles with scientometrics approach. Econ Res-Ekonomska Istraživanja.

